# Public Communication of Foodborne Mycotoxin Risks on YouTube™: A Cross-Sectional Assessment of Video Quality, Reliability, and Educational Value

**DOI:** 10.3390/toxins18080347

**Published:** 2026-08-14

**Authors:** Ömer Faruk Yeşil, Ahmet Çelik

**Affiliations:** 1 Department of Food Processing (Food Technology Program), Diyarbakır Vocational School of Agriculture, Dicle University, 21280 Diyarbakır, Türkiye; 2 Department of Crop and Animal Production (Dairy and Livestock Program), Diyarbakır Vocational School of Agriculture, Dicle University, 21280 Diyarbakır, Türkiye; ahmet.celik@dicle.edu.tr

**Keywords:** mycotoxins, food toxicology, YouTube™, risk communication, food safety education, digital health information, DISCERN

## Abstract

Mycotoxins contaminate a wide range of foods and may contribute to both acute toxicity and chronic dietary exposure. Because online video increasingly shapes public interpretation of food-safety hazards, we evaluated the quality of English-language YouTube™ content on foodborne mycotoxin risks. Of 166 records screened for eligibility, 53 were excluded and 113 videos were analyzed. Two food-safety experts independently evaluated each video with an investigator-developed Video Content Quality (VCQ) checklist, the Global Quality Scale (GQS), and a five-item modified DISCERN instrument. The final sample comprised 77 company/commercial and 36 academic/noncommercial videos. After Holm adjustment across five source-group outcomes, company/commercial videos had higher VCQ and modified DISCERN scores (both adjusted *p* < 0.001), whereas academic/noncommercial videos had a higher interaction rate (adjusted *p* = 0.024). The GQS comparison did not meet the adjusted significance threshold (adjusted *p* = 0.066) and should not be interpreted as proof of equivalence. Video age was associated with cumulative views and likes but not with any quality score after correction. Item-level VCQ results showed that authoritative-source citation and health-effects coverage were among the least frequently awarded full-credit domains. Although the three quality measures were strongly correlated, neither interaction rate nor viewing rate was significantly associated with them. Platform engagement therefore did not serve as a reliable marker of scientific quality in this sample. Source- and region-based comparisons remain exploratory because the single-query design, broad uploader categories, and uneven source composition may have shaped the sampled videos.

## 1. Introduction

Mycotoxins can reach food before harvest, during drying and storage, or later in processing and distribution. Species of Aspergillus, Fusarium, and Penicillium are among the main producers. Foods of concern include cereals, nuts, oilseeds, spices, dried fruits, coffee, cocoa, and, when contaminated feed is involved, animal-derived products such as milk [1,2,3,4,5,6]. Control remains difficult because toxin formation depends on climate, water activity, crop stress, insect damage, post-harvest handling, and storage conditions. Consumers face a further problem: contamination is not always visible, evenly distributed, or removable at home.

These compounds matter to public health because exposure can be short and intense, or repeated at lower levels over time. Acute mycotoxicosis may involve gastrointestinal illness, liver injury, immune dysfunction, and, in severe events, death. Chronic exposure is less visible in daily life, but it can contribute to carcinogenic, hepatotoxic, nephrotoxic, immunotoxic, estrogenic, developmental, or reproductive effects, depending on the compound, dose, duration, and vulnerability of the exposed group [1,2,3,4,5,6]. Naturally occurring aflatoxins are established human carcinogens, and aflatoxin B1 remains the best-known and most toxic member of that group [3,4]. Ochratoxin A, fumonisins, deoxynivalenol, zearalenone, patulin, and emerging mycotoxins add further concerns for kidney, liver, gastrointestinal, immune, endocrine, and reproductive health [1,2,5,6].

The effects of food processing are frequently misunderstood. Heat treatment, washing, scraping, or cutting away visibly mouldy portions cannot be offered as reliable safeguards for every contaminated food. Reduction depends on the toxin, food matrix, moisture, temperature, pH, processing time, and starting contamination level [7]. A clean-looking food is not automatically toxin-free, and visible mould is not a direct measure of toxin concentration. Prevention, proper drying, storage control, sorting, regulatory monitoring, and laboratory testing remain central to managing the risk.

Communication is part of that risk management. Surveillance, maximum limits, and analytical confirmation provide the technical basis for control, yet consumers decide what to buy, store, cook, discard, or eat. Those decisions are shaped by beliefs about mouldy foods, high-risk commodities, cooking, washing, detoxification claims, traditional products, and home storage. Poorly framed messages can lead to unnecessary fear and food waste; they can also create false reassurance and continued consumption of unsafe food. Useful public explanations need to stay practical without blurring the toxicological distinctions that matter.

YouTube™ is now one of the places where these messages are sought, shortened, and shared. The platform can help learning through visual explanation, expert commentary, demonstrations, news coverage, and public discussion. It also hosts promotion, personal interpretation, and content that has not passed through peer review. That mixture is consequential for mycotoxins, a technical and largely invisible hazard that is often confused with ordinary spoilage and closely tied to storage and food-preparation behaviour.

Work on other food-safety and health topics shows why structured assessment is needed. Li et al. found that many food-poisoning videos provided incomplete information [8]. Basch et al. reported that prostate-cancer videos varied substantially in content and source transparency [9]. Loeb et al. similarly showed that popular prostate-cancer videos could contain biased or misleading material [10]. Subsequent studies have applied structured quality instruments across food allergy, nutrition, complementary feeding, oral health, and other health-video topics [11,12,13,14,15,16,17,18,19]. Engagement metrics should therefore not be read as measures of scientific validity.

TikTok, Instagram Reels, YouTube Shorts, and other short-form video services are also increasingly important health-communication channels. Their rapid, algorithmically curated formats create distinct opportunities for reach and distinct risks for context loss and misinformation [20]. This study was restricted to YouTube because the prespecified protocol required stable public watch URLs, persistent upload and engagement metadata, a mixture of short- and long-form educational content, and direct comparability with the established YouTube™ quality-assessment literature. Pooling platforms with different recommendation systems, video-length conventions, metadata definitions, and search interfaces would have required separate platform-specific sampling frames and was beyond the defined cross-sectional design.

In food toxicology, visibility is a poor shortcut for judgement. A video may circulate widely because it is short, visually polished, commercially appealing, or easy to share, while still saying little about exposure route, dose, duration, matrix effects, or toxin persistence. Foodborne mycotoxins require more than a general warning about mould. A useful explanation separates fungal growth from toxin contamination, acute intoxication from chronic dietary exposure, and household handling from laboratory confirmation. It also covers major toxin groups, susceptible commodities, prevention, storage, processing stability, and the limits of unsupported cleaning or detoxification claims.

Despite this broader evidence base, we did not identify a prior video-level study focused specifically on public communication of foodborne mycotoxin risks. Accordingly, “understudied” denotes the absence of a directly comparable mycotoxin-focused quality assessment, not an absence of related food-safety or health-video research. Reliability assessment in this field commonly draws on the original DISCERN framework [21] and rationally shortened video adaptations [22]. A recent ruminal-acidosis study further illustrates the expansion of video-quality research toward mixed human and artificial-intelligence-supported assessment [23]. The remaining gap is relevant to a toxins-focused journal because mycotoxins are naturally produced, often chemically stable, unevenly distributed in food lots, difficult for consumers to detect, and linked to dose, duration, target organ, matrix, and exposure pathway.

This cross-sectional study examined how English-language YouTube™ videos communicate foodborne mycotoxin risks and whether audience engagement corresponds to scientific quality. Google Trends™ informed the appraisal of three candidate terms, after which ‘mycotoxin’ was selected as the sole analytic YouTube™ query because it names the overarching toxin class rather than a single toxin or a household mould phrase. The working hypothesis was that engagement indicators would not reliably reflect mycotoxin-specific completeness, educational value, or source transparency. Because a single umbrella query can influence which uploaders and content types are retrieved, source-group and regional comparisons were treated as exploratory. Eligibility screening covered 166 video records; 53 were excluded and 113 entered the structured assessment.

## 2. Results

### 2.1. Identification, Screening, and Inclusion of Videos

**Figure 1:**
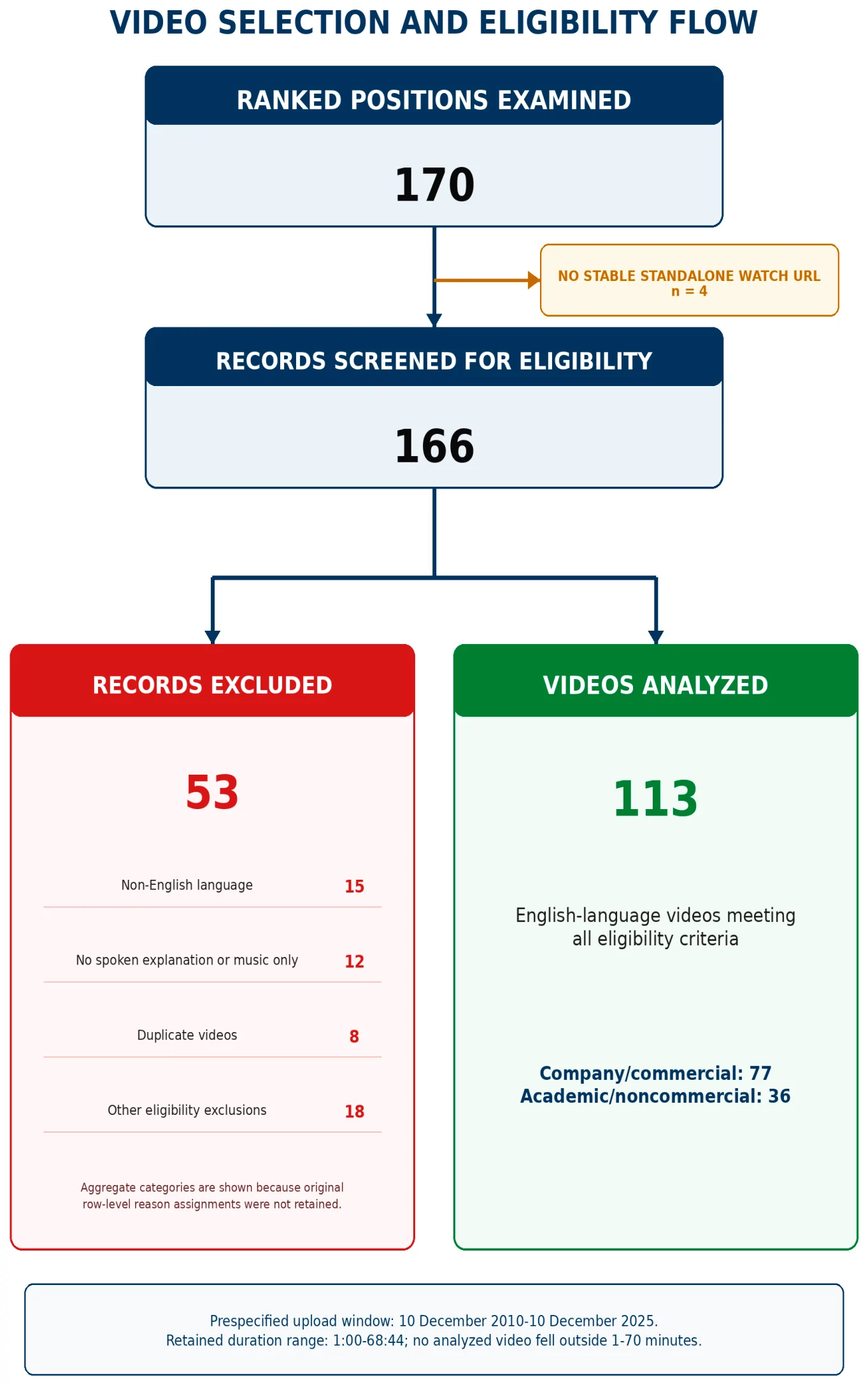
Video selection and eligibility flow. Under the prespecified protocol, the first 170 ranked positions were examined for the analytic query ‘mycotoxin’; four lacked a stable standalone watch URL. The remaining 166 records were screened, 53 records were excluded, and 113 videos were analyzed. Supplementary File S1 documents the complete ranked-position audit.

The prespecified search protocol required examination of the first 170 positions returned in YouTube™’s default relevance order for the single analytic query ‘mycotoxin’. Four positions lacked a stable standalone watch URL, so 166 video records were available for eligibility assessment. Fifty-three records were excluded and 113 videos proceeded to quantitative analysis. Eligible uploads were restricted to the 15-year period preceding the search date (10 December 2010–10 December 2025). The observed upload dates in the analyzed dataset ranged from 9 June 2011 to 1 July 2025. The retained-video duration range was 1:00–68:44, and no analyzed video was shorter than 1 min or longer than 70 min. Figure 1 presents the revised selection pathway, and Table 1 summarizes the corresponding audit counts.

### 2.2. Source Type and Geographic Origin

Company/commercial channels contributed 77 of the 113 included videos, whereas 36 were classified as academic/noncommercial. Regional coding placed 63 videos in North America, 34 in Europe, and 16 in Asia-Pacific. The source-by-region cross-tabulation in Table 2 shows that uploader composition was uneven across these geographic groups, a feature that limits region-specific interpretation.

### 2.3. Source-Group Comparisons

Table 3 reports source-group comparisons derived from the audited 113-video dataset. After Holm correction across the five outcomes, company/commercial videos had higher VCQ and modified DISCERN scores, while academic/noncommercial videos had a higher interaction rate. Adjusted comparisons for GQS and viewing rate were not significant. These results describe associations within the sampled videos and do not establish an effect of uploader type.

### 2.4. Geographic Comparisons

Table 4 presents exploratory comparisons across North America, Europe, and Asia-Pacific. After Holm adjustment across the five regional omnibus tests, VCQ, GQS, and modified DISCERN differed across regions, whereas interaction and viewing rates did not. Holm-adjusted pairwise tests showed lower scores in North America than Europe for VCQ, GQS, and modified DISCERN (all adjusted *p* < 0.001). North America also scored lower than Asia-Pacific for VCQ (adjusted *p* = 0.012) and GQS (adjusted *p* = 0.042), but not modified DISCERN (adjusted *p* = 0.289). Europe and Asia-Pacific did not differ significantly. Because Table 2 demonstrates marked source imbalance and sparse source-by-region cells, these findings are hypothesis-generating and cannot be attributed to geography itself.

### 2.5. Correlations Between Quality Scores and Engagement Metrics

Spearman correlations are shown in Table 5. VCQ, GQS, and modified DISCERN were strongly and positively correlated (rho = 0.72–0.76; all *p* < 0.001). Interaction rate and viewing rate were not significantly correlated with VCQ, GQS, modified DISCERN, or each other. Therefore, the corrected engagement measures did not function as proxies for scientific completeness, educational quality, or reliability.

### 2.6. Video Age and Its Associations

The 113 videos had been available for a mean of 1506.4 days (SD 1198.3), with a median of 1371 days (IQR 470–2065) and a range of 162–5298 days. After Holm correction across eight age correlations, video age was positively associated with cumulative views (rho = 0.475, adjusted *p* < 0.001) and likes (rho = 0.270, adjusted *p* = 0.027), but not with VCQ (rho = 0.030), GQS (rho = 0.090), modified DISCERN (rho = 0.239; adjusted *p* = 0.055), comments (rho = 0.094), interaction rate (rho = −0.244; adjusted *p* = 0.055), or viewing rate (rho = 0.029). Supplementary Table S5 provides the full distribution and correlation results. The pattern indicates that cumulative exposure metrics partly reflect time online, whereas the viewing-rate normalization remains a descriptive engagement measure and not a validated indicator of content quality.

### 2.7. Item-Level VCQ Results

**Figure 2:**
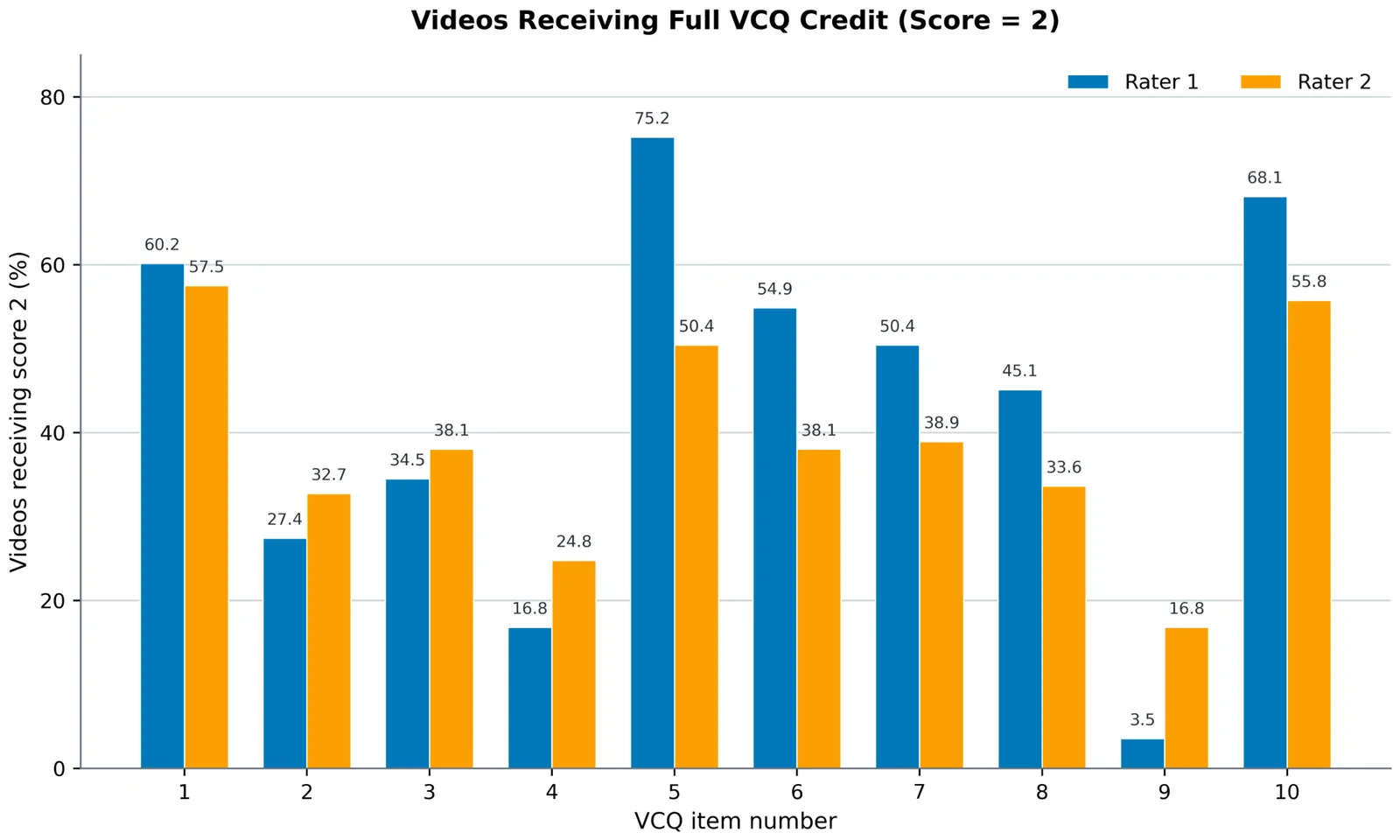
Proportion of videos receiving full VCQ credit (score = 2) for each VCQ item, shown separately for the two independent raters. Item numbers: 1, definition and scope; 2, fungal sources and ecology; 3, major mycotoxin groups; 4, health effects; 5, depth of explanation; 6, food-safety relevance; 7, analytical methods; 8, control and prevention; 9, authoritative sources; 10, evidence support. The figure was generated directly from the item-level data in Supplementary File S1.

Rater-specific item distributions are reported in Supplementary Table S6 and visualized in Figure 2. Full credit (score = 2) was awarded most frequently for depth of explanation (Item 5: 75.2% by Rater 1 and 50.4% by Rater 2) and evidence support (Item 10: 68.1% and 55.8%). It was awarded least frequently for authoritative sources (Item 9: 3.5% and 16.8%) and health effects (Item 4: 16.8% and 24.8%). The distributions are presented separately because averaging two ordinal item scores would create 0.5 and 1.5 categories that do not represent the requested 0/1/2 scale. For both raters, the sum of the ten item scores matched the corresponding video-level VCQ total in all 113 videos.

### 2.8. Inter-Rater Reliability

Table 6 summarizes inter-rater reliability for the paired instrument totals. Two-way random-effects, absolute-agreement ICCs for the mean of two raters [ICC(A,2)] were excellent for VCQ, GQS, and modified DISCERN. Bootstrap confidence intervals and exact/within-one-point agreement are reported to provide reliability information beyond a single point estimate.

## 3. Discussion

### 3.1. Principal Findings

The revised analysis characterizes 113 English-language videos retained after eligibility screening: 77 from company/commercial channels and 36 from academic/noncommercial sources. Company/commercial videos received higher VCQ and modified DISCERN scores after multiplicity correction, whereas academic/noncommercial videos showed a higher interaction rate. The adjusted GQS result (*p* = 0.066) is compatible with either a small source-group difference or no meaningful difference and should not be interpreted as definitive equivalence, particularly given the unequal group sizes. These source patterns may also reflect the single ‘mycotoxin’ query, which can preferentially retrieve technical laboratories, testing services, and commercial educational channels. The findings therefore describe this sampled query output and do not establish that commercial affiliation improves communication quality.

**Figure 3:**
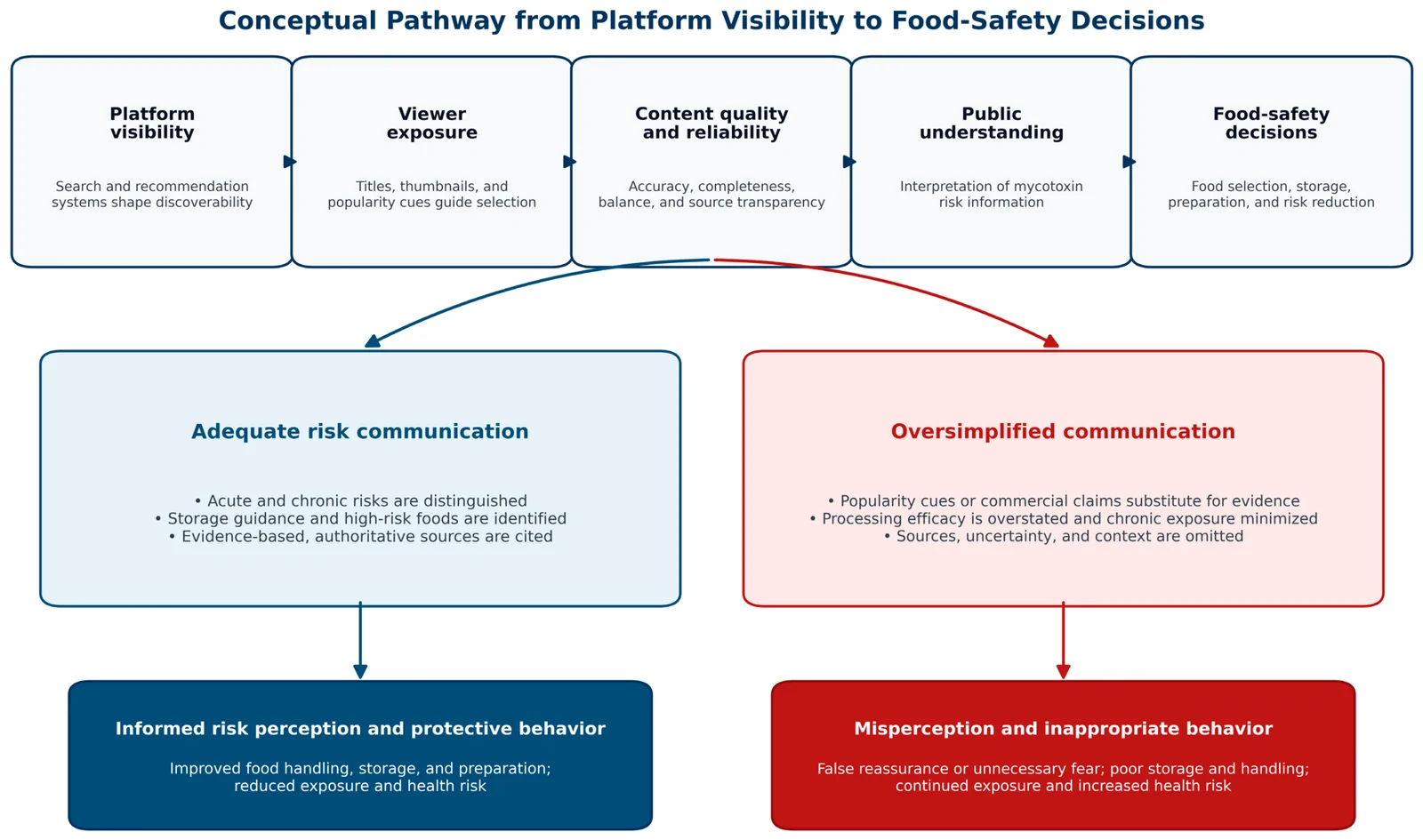
Conceptual framework for mycotoxin risk communication on YouTube™. The framework illustrates how platform visibility and viewer exposure interact with content quality and reliability, shaping public understanding and food-safety decisions. The lower pathway contrasts adequate risk communication with oversimplified messaging. Arrows indicate the direction and sequential progression of the workflow between the stages shown.

The quality instruments moved together, whereas engagement metrics followed a separate pattern. Neither interaction rate nor viewing rate was significantly associated with VCQ, GQS, or modified DISCERN. This supports the narrower conclusion that platform engagement cannot be used as a quality screen. Figure 3 presents a conceptual pathway from visibility to food-safety decisions; it is not a frequency estimate derived from the coded dataset.

### 3.2. Popularity Does Not Equal Toxicological Reliability

A widely viewed YouTube™ video is not, for that reason alone, a reliable toxicology source. Titles, thumbnails, channel size, production style, and recommendation algorithms can increase exposure without changing scientific accuracy. In this dataset, neither viewing rate nor interaction rate was significantly correlated with VCQ, GQS, or modified DISCERN. The appropriate interpretation is absence of evidence that engagement identified quality, not evidence that popular videos were systematically worse.

Table 5 supports that interpretation. The three quality measures were strongly intercorrelated, but their associations with interaction rate and viewing rate were small and non-significant. Interaction rate and viewing rate were also not significantly correlated after the rate definitions were corrected and recalculated from views, likes, comments, and days since upload.

Earlier YouTube™ studies support the need to separate engagement from quality. Li et al. reported that usefulness scores for food-poisoning videos were not correlated with likes, dislikes, views, or views per day [8]. Desai et al. found that public response to medical YouTube™ videos did not simply mirror content value [18]. Loeb et al. documented the circulation of misinformation and biased presentation [10], while Basch et al. and Madathil et al. emphasized wide variability in public-health value and reliability [9,19].

For foodborne mycotoxins, engagement-independent quality assessment remains important because toxins may be invisible, unevenly distributed within food lots, and persistent under common processing conditions. The recovered VCQ item matrices permit item-level description of the ten broad domains, but they do not contain separate subcodes for acute intoxication, chronic dietary exposure, cooking misconceptions, or individual household practices. Accordingly, the study reports only the domains actually scored and clearly labels more specific toxicological points as literature-based communication priorities.

### 3.3. Gaps in Communicating Acute and Chronic Mycotoxin Risks

A useful public explanation must separate acute intoxication from chronic dietary exposure. Acute mycotoxicosis is easier to describe because it can be linked to short-term illness and high exposure. Chronic exposure is harder to convey because it may not produce immediate symptoms, even though it remains central to food-safety risk assessment. Aflatoxins, ochratoxin A, fumonisins, deoxynivalenol, zearalenone, patulin, and other mycotoxins differ in target organs and regulatory relevance, but all require careful framing [1,2,3,4,5,6,7].

The item-level results now show that the broad health-effects domain received full credit in only 16.8% of videos for Rater 1 and 24.8% for Rater 2. However, Item 4 combined acute and chronic health effects and did not provide separate acute-versus-chronic codes. We therefore cannot estimate how often either exposure pattern was communicated. The acute/chronic discussion below is a toxicological interpretation of why this distinction matters, not a reconstructed prevalence analysis.

A balanced video should go beyond the statement that mould can be harmful. It should explain that visible mould and toxin concentration are not the same thing, that a clean-looking food is not necessarily toxin-free, and that removing the visibly mouldy part is not always protective. It should also keep immediate illness and long-term exposure clearly distinct.

### 3.4. Source Type and the Interpretation of Communication Quality

Company/commercial videos represented most of the sample and had higher adjusted VCQ and modified DISCERN scores; GQS did not meet the Holm-adjusted significance threshold. This pattern suggests that the commercial category in this particular query contained many specialized laboratories, testing services, consultancies, or technical providers capable of presenting detailed analytical and regulatory information. The single umbrella query may have contributed to that composition. This explanation is interpretive and was not separately coded; it should not be generalized to commercial health communication as a whole.

Source labels should not substitute for direct content appraisal. A commercial channel may provide technically detailed and well-sourced information, while an academic/noncommercial channel may provide only a short or general overview. Conversely, product or service promotion can still create conflicts of emphasis. The observed group differences should therefore be read as characteristics of this sample, not as a general ranking of commercial and academic communication.

Regardless of uploader type, public messages should avoid implying that cooking, washing, scraping, or removing visible mould reliably eliminates mycotoxin risk. Control and prevention (Item 8) received full credit in 45.1% of videos for Rater 1 and 33.6% for Rater 2, but the item did not distinguish accurate processing advice from each specific misconception. Several mycotoxins show considerable processing stability, and reduction depends on toxin type, food matrix, moisture, temperature, pH, processing time, and initial contamination [7]. The processing statements in this section therefore remain evidence-based recommendations rather than item-specific prevalence claims.

### 3.5. Implications for Food Toxicology Literacy and Public Health Communication

Food toxicology literacy needs more attention in online communication. Mycotoxins sit at the intersection of fungal ecology, agriculture, storage, chemistry, toxicology, laboratory analysis, and regulation. Consumers may search online after seeing mould on food, hearing about aflatoxin, or encountering a product-related claim. The information they find can influence storage, disposal, cooking, purchasing, and risk perception.

Universities, public health agencies, food-safety authorities, toxicology experts, and professional societies can use YouTube™ as part of the food-safety communication environment. Technical reports remain necessary, but many viewers first meet the topic through a short video. Those videos need plain language, visible sources, and enough toxicological detail to avoid false reassurance.

Useful videos would answer practical questions directly: Which foods are most vulnerable? Can toxins be present without visible mould? Does cooking destroy mycotoxins? When should food be discarded? When is laboratory testing needed? What do regulatory limits mean? The present VCQ captured food-safety relevance and control/prevention broadly but did not code these questions separately; they are therefore recommendations for future communication and item-level instruments rather than observed frequencies in this dataset.

### 3.6. Comparison with Previous YouTube™ Health and Food-Safety Studies

Our work adds a food-toxicology case to the video-quality literature that has mostly focused on clinical, dental, nutrition, and general food-safety topics. Li et al. found that educational videos on food poisoning were generally more useful, but usefulness did not track viewer engagement [8]. That result is close to ours: viewing and interaction metrics did not work as reliable indicators of content quality.

Basch et al. examined Escherichia coli videos and highlighted the need for professional sources to provide accurate public information [9]. Aytekin Şahin et al. reported variability in YouTube™ videos on food additives [11], and Reddy et al. found that food allergy videos required appraisal of educational quality before use as public resources [12]. Food additives, bacterial hazards, allergens, and mycotoxins are different risk categories, but all can be misunderstood when public communication is incomplete, promotional, or detached from evidence.

Structured scoring has also been useful in other video-quality studies [13,14,15,16,17,22]. The present regional analysis used a documented mapping: North America (*n* = 63), Europe (*n* = 34), and Asia-Pacific (*n* = 16). Academic/noncommercial videos represented 30/63 North American videos, compared with 3/34 European and 3/16 Asia-Pacific videos. Although quality-score omnibus tests remained significant after Holm adjustment, these findings are deliberately treated as exploratory because source mix, institutional context, language, production characteristics, and the single-query search design may explain the observed pattern. No stable geographic ranking is inferred.

### 3.7. Strengths and Limitations

The analysis has several practical strengths. It focused on a defined food-toxicology topic, followed a prespecified 15-year eligibility window and 170-position screening depth, retained canonical URLs, applied the 1–70 min duration criterion, included 113 videos, used three complementary assessment instruments, blinded raters to engagement metrics during scoring, and now provides paired rater totals and reproducible analysis code. Agreement was excellent across VCQ, GQS, and modified DISCERN.

Several limitations define the scope of these findings. YouTube™ results change over time and may vary with location; the study represents one default-relevance snapshot obtained from Türkiye with a single umbrella query, a prespecified 170-position screening depth, and a 15-year upload window. Although Google Trends™ informed appraisal of ‘mycotoxin’, ‘mould in food’, and ‘aflatoxin’, only ‘mycotoxin’ generated the analytic sample. This design may have favoured technically framed content and contributed to the dominance of company/commercial videos and the observed VCQ, GQS, and modified DISCERN patterns; videos discoverable only through consumer phrasing or toxin-specific terms were not sampled. Restriction to English also precludes linguistic and cultural generalization. No a priori power analysis was performed. With *n* = 36 versus *n* = 77, the study had about 80% power to detect d ≈ 0.57, so smaller but meaningful differences—particularly the adjusted GQS result of *p* = 0.066—could have been missed. Final consensus source labels were retained, but the number of initially discordant classifications was not, preventing retrospective source-label kappa estimation. VCQ is an investigator-developed content checklist without external psychometric validation. The recovered item matrices now support rater-specific 0/1/2 distributions, but no separate consensus item score was generated and the ten broad domains do not isolate acute versus chronic exposure, individual processing misconceptions, or each practical consumer question.

### 3.8. Recommendations for Evidence-Based Mycotoxin Risk Communication

Universities, food-safety authorities, toxicology societies, and public health institutions could produce short, visually clear, and regularly updated videos on foodborne mycotoxins. Such videos should explain acute intoxication and chronic dietary exposure separately, identify high-risk commodities, describe safe storage practices, and correct the belief that cooking or removing visible mould always removes the hazard.

Videos need traceable references. Descriptions can link to official food-safety agencies, peer-reviewed reviews, risk assessments, or university extension materials. Promotional claims should be separated from evidence-based advice, and uncertainty should be stated plainly when it affects practical decisions.

Communication should fit the audience. Consumer videos should focus on practical decisions, whereas student or professional videos can include more detail on fungal genera, toxin groups, toxicological endpoints, sampling, analytical confirmation, and regulation. Future studies should (i) determine sample size with an a priori power analysis, (ii) retain raw item-level ratings and any adjudicated consensus values as a mandatory reproducibility standard, and (iii) use prespecified multi-query designs that include umbrella, toxin-specific, spelling-variant, and consumer-language terms. Cross-platform extensions should use separate sampling frames for YouTube, TikTok, Instagram Reels, and other short-form services rather than pooling non-equivalent ranking systems.

## 4. Conclusions

The sampled YouTube™ videos differed substantially in scientific completeness, reliability, and educational usefulness. Company/commercial channels constituted the larger source group and showed higher adjusted VCQ and modified DISCERN scores, whereas academic/noncommercial videos had a higher interaction rate. The adjusted GQS result (*p* = 0.066) should be interpreted as inconclusive rather than as evidence of equivalence. These comparisons are descriptive, query-dependent, and limited to the operational categories and search snapshot used in this study.

Most importantly, neither interaction rate nor viewing rate was significantly associated with VCQ, GQS, or modified DISCERN. Older videos accumulated more views and likes, but video age was not associated with quality after multiplicity correction. Platform engagement therefore did not identify scientifically stronger videos. Item-level results highlight limited full-credit coverage of authoritative sources and health effects. Future research should use multi-query and platform-specific sampling, retain all item-level and adjudication data, and determine sample size with an a priori power analysis.

## 5. Materials and Methods

### 5.1. Study Design

We treated YouTube™ as a time-bound public information environment and examined open-access videos dealing with foodborne mycotoxin risks. The material consisted only of publicly visible audiovisual content and video-level metadata. No data were collected from human participants, animals, clinical records, private communications, or identifiable personal characteristics of people appearing in videos. Clearing the browser reduced personalization, but it could not remove YouTube™ ranking logic, location signals, recommendations, or later changes caused by new uploads and deleted videos. The dataset is therefore a snapshot of English-language search output, not a permanent or fully reproducible map of the platform.

### 5.2. Search Term Selection Using Google Trends™

Before the YouTube™ search, Google Trends™ was used on 10 December 2025 to compare ‘mycotoxin’, ‘mould in food’, and ‘aflatoxin’ under worldwide, all-category Google Web Search settings for 1 January 2015–10 December 2025. This term-appraisal window was distinct from the 15-year YouTube™ upload eligibility window and informed terminology without defining three analytic YouTube™ samples. ‘Mycotoxin’ was selected before screening because it is the taxonomic umbrella for the toxin class and was expected to retrieve content spanning multiple compounds, commodities, toxicological effects, analytical methods, and prevention strategies. ‘Aflatoxin’ denotes one major toxin group, while ‘mould in food’ is a broader household phrase that does not necessarily imply toxin contamination. The choice favoured conceptual specificity and a single reproducible query, not a claim that alternative terms were unimportant. The Google Trends™ settings and comparison are documented in Supplementary Table S1 and Figure S1.

### 5.3. YouTube™ Search Strategy

The analytic YouTube™ search was conducted from Türkiye on 10 December 2025 using the single query ‘mycotoxin’. The protocol, specified before formal screening, required assessment of the first 170 positions returned in the platform’s default relevance order and restricted eligible uploads to the preceding 15 years (10 December 2010–10 December 2025). No additional filters were applied. To reduce personalization, browser history, cache, cookies, and personalized search data were cleared as far as practicable, and the search was not performed through an account with an active viewing history. Positions 50, 135, 144, and 162 did not provide a stable standalone watch URL; accordingly, 166 records underwent eligibility screening. Supplementary File S1 lists all 170 positions, available canonical URLs, upload dates, and final screening decisions. The terms ‘mould in food’ and ‘aflatoxin’ were considered during query appraisal but did not generate separate analytic samples; consequently, there is no distribution of the 166 screened records across multiple analytic queries.

### 5.4. Eligibility Criteria

Eligibility required a publicly accessible English-language video uploaded within the prespecified 15-year window (10 December 2010–10 December 2025) that addressed mycotoxins or fungal-toxin risks in foods for human consumption, contained spoken or otherwise interpretable educational narration, lasted from 1 to 70 min inclusive, and provided sufficient material for structured assessment. Records were excluded when they were unrelated to foodborne mycotoxins; confined to feed or veterinary topics without clear human-food relevance; non-English; silent or music-only; duplicated; unavailable; shorter than 1 min; longer than 70 min; outside the upload-date window; or purely promotional without substantive educational content. After screening 166 records, 53 records were excluded and 113 videos were analyzed. The analyzed videos were uploaded between 9 June 2011 and 1 July 2025 and lasted from 1:00 to 68:44; no analyzed video fell outside the prespecified upload-date or duration range.

### 5.5. Data Extraction

For every included video, the dataset recorded its canonical URL, search rank, upload date, duration, views, likes, comments, country of origin, source group, and paired rater totals. Engagement counts were captured on 10 December 2025 and were kept separate from the scoring forms. Durations were reviewed in minute:second format before conversion to decimal minutes. Supplementary File S1 reports both the recorded display value and the decimal-minute variable, and Supplementary Table S2 provides the corresponding variable definitions and derivation rules. Interaction rate was defined as ((likes + comments)/views) × 100, and viewing rate as views/days since upload. Table 1, Table 2, Table 3, Table 4, Table 5 and Table 6 and all dependent narrative results were regenerated from the audited dataset.

### 5.6. Source Classification

Uploader source was coded using a documented dominant-identity rule. Academic/noncommercial sources included universities, research institutions, government or public-health agencies, professional/scientific societies, and identifiable educator-led channels whose principal purpose was noncommercial education. Company/commercial sources included manufacturers, private laboratories, consultancies, testing or analytical services, commercial media/platforms, product- or service-oriented channels, and uploaders whose principal purpose involved promotion, sales, or paid services. Channel descriptions, institutional websites, declared purpose, links, calls to action, and the role of products or services were considered. Ambiguous cases were independently reconsidered by both food-safety experts and resolved by discussion to a single consensus label; no third reviewer was required. The historical number of initially discordant cases and the independent pre-consensus labels were not retained and therefore are not invented or retrospectively reconstructed. No included video remained unclassified. Geographic origin was based on the uploader’s official institutional location or linked official website. Countries were grouped as North America (USA and Canada), Europe, or Asia-Pacific using the mapping in Supplementary File S1.

### 5.7. Video Content Quality Index

Scientific completeness was assessed with a mycotoxin-specific Video Content Quality (VCQ) checklist developed for this study. The ten domains were constructed a priori from recurrent elements in the food-toxicology and mycotoxin risk-assessment literature [1,2,3,4,5,6,7]: definition and scope, fungal ecology, major toxin groups, health effects, explanatory depth, food-safety relevance, analytical methods, control/prevention, authoritative sources, and evidence support. Two food-safety experts reviewed the domains for relevance, non-redundancy, and scoring clarity before independent use. Each item was scored 0 (absent or inaccurate), 1 (partial), or 2 (clear and accurate), yielding a total of 0–20. VCQ remains an investigator-developed content checklist and has not undergone external psychometric validation. Both item-level rater matrices are retained in Supplementary File S1; their sums reconcile exactly with all 113 video-level VCQ totals. Rater-specific means, standard deviations, and 0/1/2 distributions are reported in Supplementary Table S6.

### 5.8. Global Quality Scale

The Global Quality Scale (GQS) captured overall educational quality: clarity, organization, flow, usefulness, and suitability for a general audience. In the scoring scheme, GQS complemented VCQ. VCQ measured topic-specific scientific completeness, whereas GQS reflected how understandable and useful the video was for viewers. The scale has been used in several YouTube™ health-information studies for that purpose [12,13,16,17,22].

### 5.9. Modified DISCERN Reliability Assessment

We assessed reliability with a five-item modified DISCERN instrument. The original 16-item DISCERN was designed for written consumer information about treatment choices [21], so items that presuppose a treatment-decision document were not directly transferable to short audiovisual risk communication. We therefore selected five constructs commonly retained in video adaptations and directly observable across food-safety videos: clarity of aims, use of reliable and traceable sources, balance and impartiality, signposting of additional information, and acknowledgment of uncertainty [22]. Each construct was scored 0 (no) or 1 (yes), producing a 0–5 total. This rational adaptation was intended to assess source transparency and communication reliability; it was not presented as a psychometric validation of the original DISCERN instrument. The exact operational wording is reported in Table 7 and Supplementary Table S3.

### 5.10. Evaluation Procedure and Inter-Rater Reliability

Two food-safety experts evaluated all included videos independently and watched each video in full. Both had experience in food microbiology, food toxicology, and mycotoxin risk communication. VCQ, GQS, and modified DISCERN forms were completed without access to engagement metrics. The mean of the two raters’ total scores was used in descriptive and inferential analyses. Reliability was assessed using a two-way random-effects, absolute-agreement ICC for the mean of two raters [ICC(A,2)]. Percentile 95% confidence intervals were obtained from 10,000 video-level bootstrap samples, and exact and within-one-point agreement were also reported. Supplementary Table S4 provides the complete scoring procedure and interpretation guide.

### 5.11. Statistical Analysis

Analyses were performed in R, version 4.3.2 (R Foundation for Statistical Computing, Vienna, Austria) [24], and independently checked using Microsoft Excel 2019 (Microsoft Corporation, Redmond, WA, USA), included in Microsoft Office Professional 2019. Continuous variables were summarized with mean, standard deviation where requested, median, interquartile range where requested, minimum, and maximum; categorical variables were summarized by frequency and percentage. Two-sided Mann–Whitney U tests compared source groups, Kruskal–Wallis tests compared regions, and Holm adjustment controlled multiplicity across the five source-group outcomes and five regional omnibus tests. Significant regional quality outcomes were followed by Holm-adjusted pairwise tests. Spearman’s rho assessed associations among quality measures and engagement metrics. A separate eight-test Holm family assessed video age against VCQ, GQS, modified DISCERN, views, likes, comments, interaction rate, and viewing rate. No a priori power analysis was performed. The unequal source groups (*n* = 36 and *n* = 77) provided approximately 80% power to detect d ≈ 0.57 at two-sided alpha = 0.05; smaller effects could be missed. Future confirmatory studies should determine sample size a priori for the smallest effect considered scientifically meaningful.

## Figures and Tables

**Table 1:** Video selection and eligibility screening.

Screening Step	*n*	Description
Ranked result positions examined	170	Prespecified screening depth for the single analytic query ‘mycotoxin’
Positions without a stable standalone watch URL	4	Listed individually in the screening log
Video records screened	166	Stable watch URLs captured for eligibility screening
Videos excluded	53	Excluded according to eligibility criteria
Videos analyzed	113	English-language videos meeting all eligibility criteria
Retained videos longer than 70 min	0	The maximum audited duration was 68:44
Retained videos shorter than 1 min	0	The minimum audited duration was exactly 1:00
Non-English language	15	Aggregate exclusion reason
No spoken explanation/only background music	12	Aggregate exclusion reason
Duplicate videos	8	Aggregate exclusion reason
Other eligibility exclusions	18	Residual aggregate because original row-level reasons were not retained
**Total excluded**	**53**	**All aggregate exclusion categories combined**

Note: The prespecified search protocol set the screening depth at the first 170 positions returned in default relevance order for the single analytic query. Four positions did not produce a stable standalone watch URL and are listed individually in Supplementary File S1. Eligible uploads were limited to the 15-year period preceding the search date, and no included video was below 1 min or above 70 min.

**Table 2:** Cross-tabulation of included videos by source group and geographic region.

Region	Academic/Noncommercial	Company/Commercial	Total
North America	30	33	63
Europe	3	31	34
Asia-Pacific	3	13	16
**Total**	**36**	**77**	**113**

Note: North America comprised the USA and Canada; Europe comprised Austria, Belgium, Denmark, France, Germany, Greece, Ireland, Italy, the Netherlands, Serbia, Switzerland, and the UK; Asia-Pacific comprised Australia, China, India, and Japan. All 113 included videos had an assignable country and final consensus source code.

**Table 3:** Comparison of quality and engagement metrics between source groups.

Parameter	Source	*n*	Mean	Median	Min	Max	Raw *p*/Holm-Adjusted *p*
VCQ	Company/commercial	77	13.16	14.50	1.00	20.00	<0.001/<0.001
VCQ	Academic/noncommercial	36	9.79	9.75	3.00	16.00	
GQS	Company/commercial	77	3.86	4.00	1.00	5.00	0.033/0.066
GQS	Academic/noncommercial	36	3.60	4.00	2.00	5.00	
Modified DISCERN	Company/commercial	77	4.03	4.00	0.00	5.00	<0.001/<0.001
Modified DISCERN	Academic/noncommercial	36	3.00	3.00	2.00	5.00	
Interaction rate (%)	Company/commercial	77	2.42	1.39	0.00	31.46	0.008/0.024
Interaction rate (%)	Academic/noncommercial	36	3.64	3.43	0.00	12.96	
Viewing rate (views/day)	Company/commercial	77	3.65	0.28	0.02	185.25	0.072/0.072
Viewing rate (views/day)	Academic/noncommercial	36	6.95	0.54	0.02	84.82	

Note: Values are expressed as mean, median, minimum, and maximum. Source-group comparisons used two-sided Mann–Whitney–U tests; the *p*-value column reports raw *p*/Holm-adjusted *p* across the five outcomes. Interaction rate = ((likes + comments)/views) × 100; viewing rate = views/days since upload. Analytical VCQ, GQS, and modified DISCERN scores were the means of the two raters’ totals. VCQ: Video Content Quality; GQS: Global Quality Scale.

**Table 4:** Quality and engagement comparisons by geographic region.

Parameter	Region	*n*	Mean	Median	Min	Max	Raw *p*/Holm-Adjusted *p*
VCQ	North America	63	10.64	12.00	1.00	17.00	<0.001/0.001
VCQ	Europe	34	13.90	15.00	6.00	20.00	
VCQ	Asia-Pacific	16	13.91	16.00	4.00	19.00	
GQS	North America	63	3.51	4.00	1.00	5.00	<0.001/0.002
GQS	Europe	34	4.13	4.00	3.00	5.00	
GQS	Asia-Pacific	16	4.06	4.25	2.00	5.00	
Modified DISCERN	North America	63	3.42	3.50	0.00	5.00	0.002/0.005
Modified DISCERN	Europe	34	4.15	4.50	2.00	5.00	
Modified DISCERN	Asia-Pacific	16	3.88	4.50	2.00	5.00	
Interaction rate (%)	North America	63	3.18	1.99	0.00	31.46	0.271/0.331
Interaction rate (%)	Europe	34	1.72	1.32	0.00	11.11	
Interaction rate (%)	Asia-Pacific	16	3.70	2.70	0.00	11.63	
Viewing rate (views/day)	North America	63	6.99	0.33	0.02	185.25	0.166/0.331
Viewing rate (views/day)	Europe	34	1.63	0.38	0.06	13.17	
Viewing rate (views/day)	Asia-Pacific	16	2.25	0.14	0.02	27.41	

Note: Values are expressed as mean, median, minimum, and maximum. Omnibus comparisons used Kruskal–Wallis–tests; the *p*-value column reports raw *p*/Holm-adjusted *p* across the five outcomes. Pairwise Mann–Whitney–U tests were Holm-adjusted within each quality measure and are reported in the text and Supplementary File S1. Regional analyses remain exploratory because source composition was uneven.

**Table 5:** Spearman correlations between quality scores and engagement metrics.

Variables	VCQ	GQS	Modified DISCERN	Interaction Rate	Viewing Rate
VCQ	1	0.76 **	0.74 **	−0.11	−0.14
GQS	0.76 **	1	0.72 **	−0.02	−0.01
Modified DISCERN	0.74 **	0.72 **	1	−0.16	−0.06
Interaction rate	−0.11	−0.02	−0.16	1	0.14
Viewing rate	−0.14	−0.01	−0.06	0.14	1

Note: Spearman’s rho was calculated using the mean of the two raters’ instrument totals. Interaction rate = ((likes + comments)/views) × 100; viewing rate = views/days since upload. VCQ: Video Content Quality; GQS: Global Quality Scale. ** *p* < 0.001.

**Table 6:** Inter-rater reliability of video scoring instruments.

Instrument	ICC(A,2)	95% Bootstrap CI	Exact Agreement	Within 1 Point	Interpretation
VCQ	0.996	0.993–0.998	98 (86.7%)	107 (94.7%)	Excellent
GQS	0.973	0.949–0.989	104 (92.0%)	113 (100.0%)	Excellent
Modified DISCERN	0.982	0.967–0.993	104 (92.0%)	113 (100.0%)	Excellent

Note: ICC(A,2): two-way random-effects, absolute-agreement ICC for the mean of two raters. Confidence intervals are percentile intervals from 10,000 video-level bootstrap samples (seed 4459061). ‘Within 1 point’ refers to the absolute difference between paired rater totals. VCQ: Video Content Quality; GQS: Global Quality Scale.

**Table 7:** Integrated scoring framework for VCQ, GQS, and modified DISCERN assessment.

Instrument	Item/Level	Criterion	Operational Definition	Score
VCQ	1	Definition and scope of mycotoxins	Defines mycotoxins clearly and scientifically as toxic fungal metabolites relevant to food safety.	0–2
VCQ	2	Fungal sources and ecology	Explains relevant fungal genera, including Aspergillus, Penicillium, and Fusarium, with appropriate examples or conditions of toxin formation.	0–2
VCQ	3	Major mycotoxin groups	Correctly classifies major toxin groups such as aflatoxins, ochratoxin A, fumonisins, deoxynivalenol, zearalenone, and patulin where relevant.	0–2
VCQ	4	Health effects	Presents health effects on the basis of scientific evidence, including acute and chronic toxicological outcomes where appropriate.	0–2
VCQ	5	Depth of explanation	Goes beyond basic statements and provides enough detail to support understanding of foodborne mycotoxin risk.	0–2
VCQ	6	Food-safety relevance	Emphasizes the importance of mycotoxins for food safety, public health, susceptible commodities, and exposure prevention.	0–2
VCQ	7	Analytical methods	Mentions analytical or screening methods for mycotoxin detection, such as ELISA, HPLC, LC-MS/MS, or related approaches.	0–2
VCQ	8	Control and prevention	Discusses control, prevention, reduction, or detoxification approaches in an evidence-based and non-misleading manner.	0–2
VCQ	9	Authoritative sources	Identifies authoritative or official sources, such as FAO, WHO, EFSA, national food-safety authorities, or food codex regulations.	0–2
VCQ	10	Evidence support	Supports claims with the scientific literature, official data, or recognized expert sources rather than opinion or promotional content.	0–2
GQS	1	Very poor quality	Provides very limited information and little or no educational value for viewers.	1
GQS	2	Poor quality	Contains incomplete, superficial, or poorly organized information; several important aspects are missing.	2
GQS	3	Moderate quality	Provides some basic information, but detail, depth, or organization remains insufficient.	3
GQS	4	Good quality	Presents the topic accurately and adequately in general terms and is useful for most viewers.	4
GQS	5	Excellent quality	Is comprehensive, clear, reliable, well structured, and highly useful for the intended audience.	5
Modified DISCERN	1	Aim clarity	Clearly states or clearly implies the aims or educational purpose of the video.	0/1
Modified DISCERN	2	Source reliability	Uses reliable, traceable, or authoritative information sources.	0/1
Modified DISCERN	3	Balance and impartiality	Presents information in a balanced, neutral, and non-promotional manner.	0/1
Modified DISCERN	4	Additional sources	Lists or clearly signposts additional sources of information.	0/1
Modified DISCERN	5	Uncertainty and limitations	Acknowledges uncertain points, limits of current evidence, or knowledge gaps where relevant.	0/1

Note: VCQ: Video Content Quality; GQS: Global Quality Scale. VCQ items were scored as 0 = absent or inaccurate, 1 = partially addressed, and 2 = clearly and accurately addressed; total VCQ scores ranged from 0 to 20. GQS was scored from 1 to 5. Modified DISCERN items were scored as 0 = no and 1 = yes; total modified DISCERN scores ranged from 0 to 5.

## Data Availability

The original contributions presented in this study are included in the article and Supplementary File S1. The supplementary workbook provides the prespecified 170-position search audit, canonical URLs, upload dates, duration values, raw engagement counts, final source and regional codes, paired VCQ/GQS/modified DISCERN totals, complete rater-specific VCQ item scores, derived-variable formulas, and reproducibility checks. The videos were publicly available at the time of collection. The historical count of initially discordant source classifications and independent pre-consensus source labels was not retained and was not reconstructed. Further inquiries can be directed to the corresponding author.

## Supplementary Materials

The following supporting information can be downloaded at: https://www.mdpi.com/article/10.3390/toxins18080347/s1, Figure S1: Google Trends–Supported Query Appraisal and Analytic YouTube Search Audit; Table S1: Search Strategy and Eligibility Screening; Table S2: Video-Level Dataset and Engagement Metrics—Variable Dictionary; Table S3: Integrated Scoring Framework for VCQ, GQS, and Modified DISCERN; Table S4: Scoring Procedure and Interpretation Guide; Table S5: Video-Age Distribution and Correlation Analysis; Table S6: Item-Level VCQ Score Distributions by Rater; File S1: Complete Search Audit, Video-Level Dataset, Paired Rater Scores, and Item-Level VCQ Data.
