# Supplementary material for: Public Communication of Foodborne Mycotoxin Risks on YouTube™: A Cross-Sectional Assessment of Video Quality, Reliability, and Educational Value
Source: Toxins (Basel). 2026 Aug 14;18(8):347. doi: 10.3390/toxins18080347 (PMC13517528; doi:10.3390/toxins18080347)
Supplement: Supplementary file 1 [file toxins-18-00347-s001.zip › Figure and Tables.pdf]

Supplementary Materials: Public Communication of Foodborne Mycotoxin Risks on YouTube™: A Cross-Sectional Assessment of Video Quality, Reliability, and Educational Value

Ömer Faruk Yeşil and Ahmet Çelik

Table S1. Search Strategy and Eligibility Screening

Field	Specification	Operational note
Platform	YouTube™	Publicly available videos were screened.
Search date	10 December 2025	All metadata were captured on the same date.
Language	English	Language was assessed from spoken content.
Analytic query	mycotoxin	Google Trends candidates appraised worldwide (Google Web Search, all categories): mycotoxin; mould in food; aflatoxin, 1 January 2015–10 December 2025.
Search filters	No additional filters	Default-relevance ordering was used in a cleared browser environment.
Duration eligibility	1:00–70:00 minutes	Operational range used for the final screening dataset.
Final included sample	113	Records retained for structured analysis.
**Screening step**	**n**	**Description**
Ranked result positions examined	170	Prespecified screening depth for the single analytic query 'mycotoxin'.
Positions without stable standalone watch URL	4	Search ranks 50, 135, 144, and 162; retained in Screening_Log for auditability.
Video records screened	166	Stable watch URLs available for eligibility assessment.
Videos excluded	53	Excluded according to the eligibility criteria.
Videos included in final analysis	113	Publicly available English-language videos suitable for structured assessment.
Non-English language	15	Aggregate exclusion category.
No spoken explanation / only background music	12	Aggregate exclusion category.
Duplicate videos	8	Aggregate exclusion category.
Other eligibility exclusions	18	Residual aggregate category; row-level subassignments were not retained.
Total excluded	53	15 + 12 + 8 + 18 = 53.

Table S2. Video-Level Dataset and Engagement Metrics — Variable Dictionary

Variable	Type	Definition	Allowed values / unit	Derivation
Record_ID	Integer	Sequential record identifier	1–113	Assigned sequentially
Video_SN	Integer	Original screening serial number	Original identifier	Preserved
URL	Text	Public YouTube™ video URL	URL	Preserved; malformed link repaired from the matching screening record
Source_Type	Categorical	Uploader source classification		Standardized to the exact manuscript terminology
Country	Categorical	Recorded country of origin	Country	Preserved
Continent	Categorical	Geographic grouping	North America; Europe; Asia-Pacific	Country-to-region mapping: USA and Canada = North America; Australia, China, India, and Japan = Asia-Pacific
Views	Integer	Visible view count at extraction	Count	Preserved
Likes	Integer	Visible like count at extraction	Count	Preserved
Comments	Integer	Visible comment count at extraction	Count	Preserved
Upload_Date	Date	Video upload date	YYYY-MM-DD	Standardized from original date text
Days_Since_Upload	Integer	Days from upload date to 10 December 2025	Days	DATE(2025,12,10) − Upload_Date
Duration_As_Recorded	Text	Duration value as displayed by YouTube at extraction	mm:ss or h:mm:ss text	Preserved as displayed text
VCQ_Rater_1 / VCQ_Rater_2	Integer	Independent Video Content Quality totals	0–20	Preserved raw rater totals
VCQ_Mean	Numeric	Arithmetic mean of the two VCQ totals	0–20	AVERAGE(VCQ_Rater_1, VCQ_Rater_2)
GQS_Rater_1 / GQS_Rater_2	Integer	Independent Global Quality Scale totals	1–5	Preserved raw rater totals
GQS_Mean	Numeric	Arithmetic mean of the two GQS totals	1–5	AVERAGE(GQS_Rater_1, GQS_Rater_2)
Modified_DISCERN_Rater_1 / Rater_2	Integer	Independent modified DISCERN totals	0–5	Preserved raw rater totals
Modified_DISCERN_Mean	Numeric	Arithmetic mean of the two modified DISCERN totals	0–5	AVERAGE(two rater totals)
Interaction_Rate_Pct	Numeric	Visible interactions relative to views	%	((Likes + Comments) / Views) × 100
Viewing_Rate_Views_Per_Day	Numeric	Mean views accumulated per day since upload	Views/day	Views / Days_Since_Upload
VCQ_Item_1–10_Rater_1 / Rater_2	Integer	Independent item-level VCQ ratings	0, 1, or 2	Preserved in S7; rater-specific summaries in S6
VCQ_Item_Sum_Check	Text	Audit of item-level total against reported VCQ total	OK / CHECK	Formula-driven in S7
Duration_Minutes	Numeric	Video duration expressed in decimal minutes	Minutes	Formula-derived from Duration_As_Recorded (mm:ss or h:mm:ss)

Table S3. Integrated Scoring Framework for VCQ, GQS, and Modified DISCERN

Instrument	Item/level	Criterion	Operational definition	Score
VCQ	1	Definition and scope of mycotoxins	Defines mycotoxins clearly and scientifically as toxic fungal metabolites relevant to food safety.	0–2
VCQ	2	Fungal sources and ecology	Explains relevant fungal genera, including Aspergillus, Penicillium, and Fusarium, with appropriate examples or conditions of toxin formation.	0–2
VCQ	3	Major mycotoxin groups	Correctly classifies major toxin groups such as aflatoxins, ochratoxin A, fumonisins, deoxynivalenol, zearalenone, and patulin where relevant.	0–2
VCQ	4	Health effects	Presents health effects on the basis of scientific evidence, including acute and chronic toxicological outcomes where appropriate.	0–2
VCQ	5	Depth of explanation	Goes beyond basic statements and provides enough detail to support understanding of foodborne mycotoxin risk.	0–2
VCQ	6	Food-safety relevance	Emphasizes the importance of mycotoxins for food safety, public health, susceptible commodities, and exposure prevention.	0–2
VCQ	7	Analytical methods	Mentions analytical or screening methods for mycotoxin detection, such as ELISA, HPLC, LC-MS/MS, or related approaches.	0–2
VCQ	8	Control and prevention	Discusses control, prevention, reduction, or detoxification approaches in an evidence-based and non-misleading manner.	0–2
VCQ	9	Authoritative sources	Identifies authoritative or official sources, such as FAO, WHO, EFSA, national food-safety authorities, or food codex regulations.	0–2
VCQ	10	Evidence support	Supports claims with scientific literature, official data, or recognized expert sources rather than opinion or promotional content.	0–2
GQS	1	Very poor quality	Provides very limited information and little or no educational value for viewers.	1
GQS	2	Poor quality	Contains incomplete, superficial, or poorly organized information; several important aspects are missing.	2
GQS	3	Moderate quality	Provides some basic information, but detail, depth, or organization remains insufficient.	3
GQS	4	Good quality	Presents the topic accurately and adequately in general terms and is useful for most viewers.	4
GQS	5	Excellent quality	Is comprehensive, clear, reliable, well structured, and highly useful for the intended audience.	5
Modified DISCERN	1	Aim clarity	The aims or educational purpose are clear and achieved.	0/1
Modified DISCERN	2	Source reliability	Reliable and traceable information sources are used.	0/1
Modified DISCERN	3	Balance and impartiality	Information is presented in a balanced and unbiased manner.	0/1
Modified DISCERN	4	Additional sources	Additional sources of information are listed or clearly signposted.	0/1
Modified DISCERN	5	Uncertainty	Areas of uncertainty or limitations of the evidence are acknowledged.	0/1

Table S4. Scoring Procedure and Interpretation Guide

Section	Procedure	Scale / interpretation	Quality-control rule
Blinding	Engagement metrics were stored outside scoring forms and were unavailable to evaluators during content assessment.	Not applicable	Prevents popularity cues from influencing scoring
Independent assessment	Two food-safety experts watched and scored every included video independently.	Two rater-specific totals retained	Do not overwrite raw rater scores
VCQ	Ten topic-specific domains were scored for scientific completeness and accuracy.	0 = absent/inaccurate; 1 = partial; 2 = clear/accurate; total 0–20	Use the full framework in Table S3
GQS	Overall educational quality, clarity, organization, flow, and usefulness were assessed.	1 = very poor to 5 = excellent	Rate overall viewer usefulness
Modified DISCERN	Five reliability criteria were assessed.	0 = no; 1 = yes; total 0–5	Apply the established five criteria: clear aims, reliable sources, balance, additional sources, and uncertainty
Rater mean	Arithmetic mean of the two independent rater totals is calculated for transparency.	Two decimal places	Use the same analysis score definition consistently in the manuscript and supplementary dataset
Inter-rater reliability	ICC should be reported with model, type, and unit explicitly stated.	<0.50 poor; 0.50–0.75 moderate; 0.75–0.90 good; >0.90 excellent	Specify whether single-measure or average-measure ICC was used
Engagement	Interaction rate and viewing rate must be calculated from visible metadata using the formulas in Table S2.	Interaction rate: %; viewing rate: views/day	Do not substitute raw likes for interaction rate or views per 100 days for views/day

Table S5. Video-Age Distribution and Correlation Analysis

Video-age distribution	n	Mean (SD)	Median (IQR)	Min–Max
Days since upload	113	1506.4 (1198.3)	1371 (470–2065); IQR = 1595	162–5298

Outcome	n	Spearman ρ	Raw p	Holm-adjusted p
VCQ	113	0,030	0,752	1,000
GQS	113	0,090	0,340	1,000
Modified DISCERN	113	0,239	0,011	0,055
Views	113	0,475	<0.001	<0.001
Likes	113	0,270	0,004	0,027
Comments	113	0,094	0,323	1,000
Interaction rate (%)	113	-0,244	0,009	0,055
Viewing rate (views/day)	113	0,029	0,758	1,000

**Method**	Spearman correlations used Days_Since_Upload and the corresponding outcome in
**Multiplicity**	The eight two-sided p-values were adjusted as one family using the Holm procedure.
**Interpretation**	Video age was associated with cumulative views and likes but not with any quality score after Holm
**Item-level data**	The complete rater-specific VCQ item matrices and total-score audit are retained in S7; distributions are reported in S6.
**Source adjudication**	The final consensus source labels are retained. The historical count of initially discordant source classifications was not retained and was not reconstructed.

Table S6. Item-Level VCQ Score Distributions by Rater

Rater 1 (n = 113 videos)									
**Item**	**VCQ domain**	**Mean**	**SD**	**Score 0, n**	**Score 0, %**	**Score 1, n**	**Score 1, %**	**Score 2, n**	**Score 2, %**
1	Definition and scope of mycotoxins	1,55	#AD?	6	5,3%	39	34,5%	68	60,2%
2	Fungal sources and ecology	1,02	#AD?	29	25,7%	53	46,9%	31	27,4%
3	Major mycotoxin groups	1,19	#AD?	18	15,9%	56	49,6%	39	34,5%
4	Health effects	0,77	#AD?	45	39,8%	49	43,4%	19	16,8%
5	Depth of explanation	1,72	#AD?	4	3,5%	24	21,2%	85	75,2%
6	Food-safety relevance	1,40	#AD?	17	15,0%	34	30,1%	62	54,9%
7	Analytical methods	1,29	#AD?	24	21,2%	32	28,3%	57	50,4%
8	Control and prevention	1,20	#AD?	28	24,8%	34	30,1%	51	45,1%
9	Authoritative sources	0,39	#AD?	73	64,6%	36	31,9%	4	3,5%
10	Evidence support	1,63	#AD?	6	5,3%	30	26,5%	77	68,1%

Rater 2 (n = 113 videos)									
**Item**	**VCQ domain**	**Mean**	**SD**	**Score 0, n**	**Score 0, %**	**Score 1, n**	**Score 1, %**	**Score 2, n**	**Score 2, %**
1	Definition and scope of mycotoxins	1,53	#AD?	5	4,4%	43	38,1%	65	57,5%
2	Fungal sources and ecology	1,11	#AD?	25	22,1%	51	45,1%	37	32,7%
3	Major mycotoxin groups	1,21	#AD?	19	16,8%	51	45,1%	43	38,1%
4	Health effects	0,99	#AD?	29	25,7%	56	49,6%	28	24,8%
5	Depth of explanation	1,42	#AD?	10	8,8%	46	40,7%	57	50,4%
6	Food-safety relevance	1,25	#AD?	15	13,3%	55	48,7%	43	38,1%
7	Analytical methods	1,20	#AD?	21	18,6%	48	42,5%	44	38,9%
8	Control and prevention	1,13	#AD?	23	20,4%	52	46,0%	38	33,6%
9	Authoritative sources	0,71	#AD?	52	46,0%	42	37,2%	19	16,8%
10	Evidence support	1,47	#AD?	10	8,8%	40	35,4%	63	55,8%

**Reporting note**	The score distributions are presented separately for each evaluator. Averaging two ordinal item ratings can produce 0.5 and 1.5 and would no longer represent the requested
**Integrity check**	For both evaluators, the sum of the 10 VCQ item scores exactly matches the corresponding video-level VCQ total for all 113 videos.
**Figure note**	The chart reports the proportion of videos receiving full credit (score = 2) for each VCQ item; values are formula-linked to this table.

**VCQ item**	**Rater 1**	**Rater 2**
Item 1	60,2%	57,5%
Item 2	27,4%	32,7%
Item 3	34,5%	38,1%
Item 4	16,8%	24,8%
Item 5	75,2%	50,4%
Item 6	54,9%	38,1%
Item 7	50,4%	38,9%
Item 8	45,1%	33,6%
Item 9	3,5%	16,8%
Item 10	68,1%	55,8%

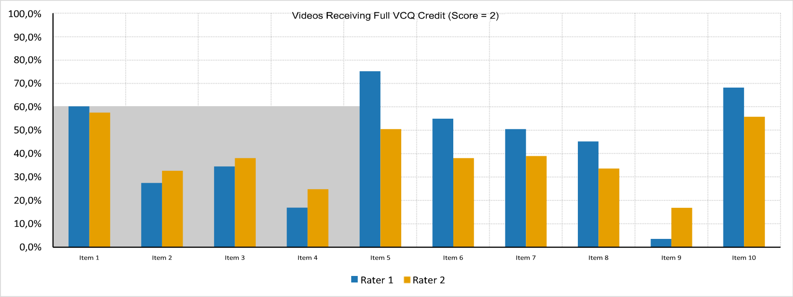

Record_ID	Video_SN	VCQ_Item_1_Rater_1	VCQ_Item_2_Rater_1	VCQ_Item_3_Rater_1	VCQ_Item_4_Rater_1	VCQ_Item_5_Rater_1	VCQ_Item_6_Rater_1	VCQ_Item_7_Rater_1	VCQ_Item_8_Rater_1	VCQ_Item_9_Rater_1	VCQ_Item_10_Rater_1	VCQ_Rater_1_Recalculated	VCQ_Rater_1_Reported	Rater_1_Total_Check	VCQ_Item_1_Rater_2	VCQ_Item_2_Rater_2	VCQ_Item_3_Rater_2	VCQ_Item_4_Rater_2	VCQ_Item_5_Rater_2	VCQ_Item_6_Rater_2	VCQ_Item_7_Rater_2	VCQ_Item_8_Rater_2	VCQ_Item_9_Rater_2	VCQ_Item_10_Rater_2	VCQ_Rater_2_Recalculated	VCQ_Rater_2_Reported	Rater_2_Total_Check	Rater_Total_Difference
1	36	0	0	1	0	2	0	2	0	0	1	6	6	OK	0	0	1	0	2	0	2	0	0	1	6	6	OK	0
2	46	2	0	0	1	1	2	2	1	0	1	10	10	OK	2	0	0	1	1	1	2	1	0	1	9	9	OK	1
3	157	2	2	2	2	2	2	1	2	0	1	16	16	OK	2	2	2	2	1	2	1	1	0	1	14	14	OK	2
4	94	2	1	1	2	1	2	0	2	0	1	12	12	OK	2	1	1	2	1	2	0	2	0	1	12	12	OK	0
5	147	1	0	0	0	2	1	2	0	0	1	7	7	OK	1	0	0	0	2	1	2	0	0	1	7	7	OK	0
6	110	1	0	1	0	2	1	2	0	0	1	8	8	OK	1	0	1	0	2	1	1	0	0	1	7	7	OK	1
7	34	1	1	1	0	2	1	0	0	0	1	7	7	OK	1	1	1	0	2	1	0	0	0	1	7	7	OK	0
8	49	2	2	2	2	2	2	0	0	0	1	13	13	OK	2	2	2	2	2	2	0	0	0	1	13	13	OK	0
9	58	2	2	1	2	1	2	0	0	1	1	12	12	OK	2	2	1	1	1	1	0	0	1	1	10	10	OK	2
10	60	1	2	1	1	1	1	1	0	0	0	8	8	OK	1	2	1	1	1	1	1	0	0	0	8	8	OK	0
11	66	1	0	1	0	2	0	2	0	0	2	8	8	OK	1	0	1	0	2	0	2	0	0	2	8	8	OK	0
12	79	1	0	1	1	0	1	0	0	0	1	5	5	OK	1	0	1	1	0	1	0	0	0	1	5	5	OK	0
13	90	2	1	1	1	1	0	2	0	0	2	10	10	OK	2	1	0	1	1	0	1	0	0	2	8	8	OK	2
14	91	2	1	1	1	2	2	0	2	0	2	13	13	OK	2	1	1	1	2	2	0	2	0	2	13	13	OK	0
15	92	0	0	0	0	2	0	1	0	0	2	5	5	OK	0	0	0	0	2	0	1	0	0	2	5	5	OK	0
16	93	2	1	1	1	2	1	2	0	0	2	12	12	OK	2	1	2	1	2	1	0	1	1	1	12	12	OK	0
17	95	1	0	1	0	2	1	2	0	0	2	9	9	OK	2	1	0	1	1	1	1	0	0	2	9	9	OK	0
18	96	0	0	0	0	2	0	1	0	0	2	5	5	OK	1	0	0	0	1	1	1	0	0	1	5	5	OK	0
19	98	1	0	1	0	2	0	1	0	0	2	7	7	OK	0	0	0	0	2	1	1	0	0	2	6	6	OK	1
20	101	1	0	0	0	1	0	0	1	0	1	4	4	OK	1	0	1	0	1	0	0	0	0	1	4	4	OK	0
21	109	2	1	1	1	2	2	2	2	0	2	15	15	OK	2	1	1	2	1	2	1	1	2	2	15	15	OK	0
22	118	2	2	1	0	2	1	0	0	0	2	10	10	OK	1	0	1	1	1	1	2	1	0	2	10	10	OK	0
23	160	2	1	2	1	2	1	2	2	0	2	15	15	OK	2	2	2	1	2	1	1	1	1	2	15	15	OK	0
24	165	2	1	2	0	2	1	0	2	0	2	12	12	OK	2	1	2	1	2	1	1	1	0	1	12	12	OK	0
25	65	2	1	2	0	2	2	2	2	0	2	15	15	OK	2	1	2	0	2	2	1	2	1	2	15	15	OK	0
26	154	2	0	1	0	2	1	0	2	0	2	10	10	OK	2	1	1	0	1	0	2	1	0	2	10	10	OK	0
27	76	1	0	1	0	2	0	2	0	0	2	8	8	OK	2	0	1	0	1	1	0	1	0	2	8	8	OK	0
28	130	1	0	0	0	1	0	0	1	0	0	3	3	OK	1	0	1	0	1	0	0	0	0	0	3	3	OK	0
29	80	2	2	2	1	2	1	2	0	1	2	15	15	OK	1	2	2	1	1	1	2	1	2	2	15	15	OK	0
30	102	2	2	1	0	1	2	0	2	0	1	11	11	OK	1	2	1	1	1	1	1	0	1	2	11	11	OK	0
31	114	1	0	0	0	2	0	2	0	0	1	6	6	OK	1	1	1	0	1	1	0	1	0	0	6	6	OK	0
32	125	1	1	1	0	1	1	0	0	0	1	6	6	OK	1	0	0	0	2	0	2	0	0	1	6	6	OK	0
33	142	2	1	2	1	2	2	2	1	1	2	16	16	OK	2	1	2	1	2	2	1	2	1	2	16	16	OK	0
34	150	2	1	2	1	1	1	0	2	0	1	11	11	OK	2	1	1	1	2	2	0	1	0	1	11	11	OK	0
35	151	2	0	2	1	2	1	0	1	0	2	11	11	OK	2	1	2	1	1	1	0	2	0	1	11	11	OK	0
36	156	2	1	2	2	2	2	1	1	1	2	16	16	OK	2	1	2	2	2	1	2	1	1	2	16	16	OK	0
37	9	1	0	1	0	1	2	1	1	0	1	8	8	OK	2	1	0	2	0	0	1	1	1	0	8	8	OK	0
38	24	1	0	1	0	2	2	2	1	0	2	11	11	OK	1	0	1	1	1	2	2	1	1	1	11	11	OK	0
39	129	2	2	2	1	2	2	2	2	0	2	17	17	OK	2	2	1	2	2	2	2	1	2	1	17	17	OK	0
40	72	1	1	0	0	0	1	0	1	0	0	4	4	OK	0	1	0	1	0	0	1	1	0	0	4	4	OK	0
41	82	1	1	0	0	0	1	1	0	0	0	4	4	OK	1	1	0	0	0	1	0	1	0	0	4	4	OK	0
42	139	2	2	2	2	2	2	2	2	1	2	19	19	OK	2	2	2	2	1	2	2	2	2	2	19	19	OK	0
43	12	2	1	1	1	1	0	1	1	0	1	9	9	OK	1	0	1	1	1	0	1	2	1	1	9	9	OK	0
44	13	2	2	2	1	2	1	2	2	1	2	17	17	OK	2	2	2	2	2	1	1	1	2	2	17	17	OK	0
45	15	2	1	1	0	2	0	2	0	1	2	11	11	OK	1	2	1	1	1	1	2	1	0	1	11	11	OK	0
46	18	2	2	2	2	2	2	2	2	2	2	20	20	OK	2	2	2	2	2	2	2	2	2	2	20	20	OK	0
47	19	2	2	2	2	2	2	2	1	1	2	18	18	OK	2	2	2	2	2	1	1	2	2	2	18	18	OK	0
48	23	2	2	2	2	2	2	2	1	1	2	18	18	OK	2	2	2	2	2	2	2	1	1	2	18	18	OK	0
49	38	2	2	1	2	1	1	0	2	1	2	14	14	OK	2	2	0	2	1	2	2	1	1	1	14	14	OK	0
50	70	2	2	2	1	2	2	1	2	0	2	16	16	OK	2	2	2	2	2	1	0	2	1	2	16	16	OK	0
51	75	2	1	1	1	2	2	2	2	1	2	16	16	OK	2	2	2	1	1	1	1	2	0	2	14	14	OK	2
52	104	1	1	1	0	2	1	1	1	1	2	11	11	OK	1	1	1	2	0	1	2	1	1	1	11	11	OK	0
53	121	1	0	1	1	2	0	1	2	1	2	11	11	OK	1	2	1	0	2	1	1	1	0	2	11	11	OK	0
54	169	2	1	1	1	2	2	2	2	1	2	16	16	OK	2	1	2	2	2	1	2	1	1	2	16	16	OK	0
55	39	2	1	1	1	2	2	2	2	1	2	16	16	OK	1	1	2	1	2	2	1	2	2	2	16	16	OK	0
56	40	2	2	2	1	2	2	2	2	1	2	18	18	OK	2	1	2	1	2	2	2	2	2	2	18	18	OK	0
57	52	2	2	2	1	2	2	1	2	1	2	17	17	OK	2	1	1	1	2	2	2	2	2	2	17	17	OK	0
58	103	1	0	0	1	2	0	2	1	0	1	8	8	OK	1	0	1	0	1	1	2	0	1	1	8	8	OK	0
59	127	2	2	2	2	2	2	1	2	1	2	18	18	OK	2	2	2	1	2	2	2	2	1	2	18	18	OK	0
60	141	2	2	2	2	2	1	2	2	1	2	18	18	OK	2	2	2	2	2	1	2	1	2	2	18	18	OK	0
61	63	2	1	1	1	2	1	2	2	0	2	14	14	OK	2	2	1	1	2	2	1	1	0	2	14	14	OK	0
62	108	2	1	2	1	2	1	2	2	1	2	16	16	OK	2	2	1	1	1	1	2	2	2	2	16	16	OK	0
63	21	2	1	1	1	1	0	0	2	0	1	9	9	OK	2	0	0	1	0	1	2	1	1	1	9	9	OK	0
64	56	2	1	1	1	2	2	2	2	1	2	16	16	OK	2	1	2	1	2	1	2	2	1	2	16	16	OK	0
65	137	2	1	2	1	2	2	1	2	0	2	15	15	OK	2	2	1	2	2	1	1	2	1	1	15	15	OK	0
66	158	2	1	2	2	2	2	2	2	1	2	18	18	OK	2	1	2	1	2	2	2	2	2	2	18	18	OK	0
67	105	2	2	2	1	2	2	2	2	1	2	18	18	OK	2	2	2	2	2	2	1	2	1	2	18	18	OK	0
68	145	2	2	2	1	2	2	2	2	1	2	18	18	OK	2	1	2	2	2	2	2	2	1	2	18	18	OK	0
69	55	2	1	1	1	2	2	1	2	1	2	15	15	OK	2	2	2	1	1	2	1	2	1	1	15	15	OK	0
70	78	2	0	0	1	1	1	1	2	0	1	9	9	OK	1	0	1	1	2	1	1	1	0	1	9	9	OK	0
71	115	2	2	2	1	2	2	1	2	0	2	16	16	OK	2	1	1	1	2	2	1	2	1	2	15	15	OK	1
72	124	2	1	1	1	2	2	2	2	1	2	16	16	OK	2	1	1	1	1	1	2	2	2	2	15	15	OK	1
73	149	2	1	0	1	1	1	0	2	0	1	9	9	OK	2	1	0	1	1	2	0	1	0	1	9	9	OK	0
74	112	2	1	2	1	2	2	1	1	1	2	15	15	OK	2	1	1	1	2	2	2	1	1	2	15	15	OK	0
75	5	2	2	2	2	2	1	2	1	2	2	18	18	OK	2	2	2	2	1	1	2	2	2	2	18	18	OK	0
76	41	2	2	1	1	2	2	2	2	1	2	17	17	OK	2	1	2	1	2	2	2	1	2	2	17	17	OK	0
77	32	2	1	2	1	2	2	2	2	1	2	17	17	OK	2	2	2	2	2	1	2	1	1	2	17	17	OK	0
78	42	2	1	2	1	2	2	2	1	2	2	17	17	OK	2	2	2	2	2	2	2	2	1	2	19	19	OK	-2
79	4	2	1	2	1	2	2	1	2	1	2	16	16	OK	2	1	2	1	2	1	2	1	1	2	15	15	OK	1
80	6	2	2	2	1	2	2	1	2	1	2	17	17	OK	2	1	2	1	2	2	2	2	1	2	17	17	OK	0
81	10	1	1	1	0	1	2	0	1	0	1	8	8	OK	1	1	0	1	1	1	1	1	0	1	8	8	OK	0
82	22	1	1	1	0	2	2	2	1	0	2	12	12	OK	1	1	1	1	1	1	1	2	1	2	12	12	OK	0
83	30	1	1	1	0	2	2	2	1	0	2	12	12	OK	2	1	1	1	1	1	1	2	1	1	12	12	OK	0
84	31	0	0	0	0	1	1	1	0	0	1	4	4	OK	1	0	0	1	1	1	0	0	0	0	4	4	OK	0
85	33	0	0	0	0	1	1	1	0	0	1	4	4	OK	0	1	0	0	0	0	1	1	0	1	4	4	OK	0
86	45	1	0	0	1	1	1	0	0	0	1	5	5	OK	1	0	1	0	0	1	0	1	0	1	5	5	OK	0
87	47	1	1	1	0	2	2	2	1	0	2	12	12	OK	1	2	1	1	1	2	1	2	0	1	12	12	OK	0
88	53	2	1	2	1	2	2	1	1	0	2	14	14	OK	2	2	1	1	1	1	2	1	2	1	14	14	OK	0
89	64	2	1	2	1	2	2	1	2	0	2	15	15	OK	2	1	2	2	1	1	1	2	1	2	15	15	OK	0
90	67	1	0	1	0	2	1	2	0	1	2	10	10	OK	1	1	1	0	2	1	1	1	0	2	10	10	OK	0
91	73	2	0	1	2	2	1	2	2	1	2	15	15	OK	2	1	2	1	2	2	1	1	1	2	15	15	OK	0
92	86	2	1	1	1	1	2	0	2	0	1	11	11	OK	2	1	2	0	1	1	1	1	0	2	11	11	OK	0
93	89	1	0	0	0	0	1	0	1	0	0	3	3	OK	1	0	1	0	0	0	0	0	1	0	3	3	OK	0
94	97	1	0	0	0	1	1	1	1	0	1	6	6	OK	1	0	0	1	1	1	0	1	0	1	6	6	OK	0
95	99	1	1	1	0	2	2	2	1	0	2	12	12	OK	2	1	1	1	1	2	1	2	0	1	12	12	OK	0
96	117	1	1	1	0	2	2	2	2	1	2	14	14	OK	2	2	2	1	1	1	1	1	1	2	14	14	OK	0
97	133	1	1	1	0	2	2	2	1	0	2	12	12	OK	1	0	1	2	1	2	1	1	2	1	12	12	OK	0
98	134	2	2	2	1	2	2	2	1	0	1	15	15	OK	1	1	2	1	2	2	2	1	1	2	15	15	OK	0
99	140	2	2	2	1	2	2	2	1	0	2	16	16	OK	1	1	1	2	2	2	2	2	1	2	16	16	OK	0
100	143	0	0	0	0	1	0	0	0	0	0	1	1	OK	1	0	0	0	0	0	0	0	0	0	1	1	OK	0
101	146	1	1	1	0	2	2	2	1	0	2	12	12	OK	2	2	1	1	1	1	1	1	0	1	11	11	OK	1
102	148	2	1	1	2	2	2	2	2	1	2	17	17	OK	2	2	2	1	2	2	2	2	0	2	17	17	OK	0
103	152	1	2	1	0	2	2	2	0	0	2	12	12	OK	2	2	1	2	2	0	1	1	1	0	12	12	OK	0
104	155	1	1	1	0	2	2	1	2	0	2	12	12	OK	1	1	1	0	2	2	2	1	0	2	12	12	OK	0
105	159	1	1	1	1	2	0	2	2	1	1	12	12	OK	1	1	1	1	2	1	1	1	1	2	12	12	OK	0
106	161	1	1	1	0	2	2	2	1	0	2	12	12	OK	1	2	1	0	2	2	2	0	0	2	12	12	OK	0
107	163	1	2	1	0	1	2	1	1	0	1	10	10	OK	1	1	1	0	1	2	1	1	0	2	10	10	OK	0
108	167	2	2	2	2	2	1	2	1	2	2	18	18	OK	1	2	2	1	2	1	2	2	1	2	16	16	OK	2
109	168	1	1	1	0	2	2	2	1	0	2	12	12	OK	2	1	1	1	1	2	1	1	1	1	12	12	OK	0
110	170	2	2	1	1	2	2	1	2	0	2	15	15	OK	1	1	2	1	1	2	1	2	2	2	15	15	OK	0
111	128	2	1	1	2	2	2	1	2	0	2	15	15	OK	2	2	2	0	2	2	1	2	0	2	15	15	OK	0
112	1	2	1	2	2	2	2	1	1	0	2	15	15	OK	1	1	2	2	2	1	1	1	1	2	14	14	OK	1
113	106	1	1	1	0	2	2	1	2	0	2	12	12	OK	1	1	1	0	2	2	2	2	0	2	13	13	OK	-1

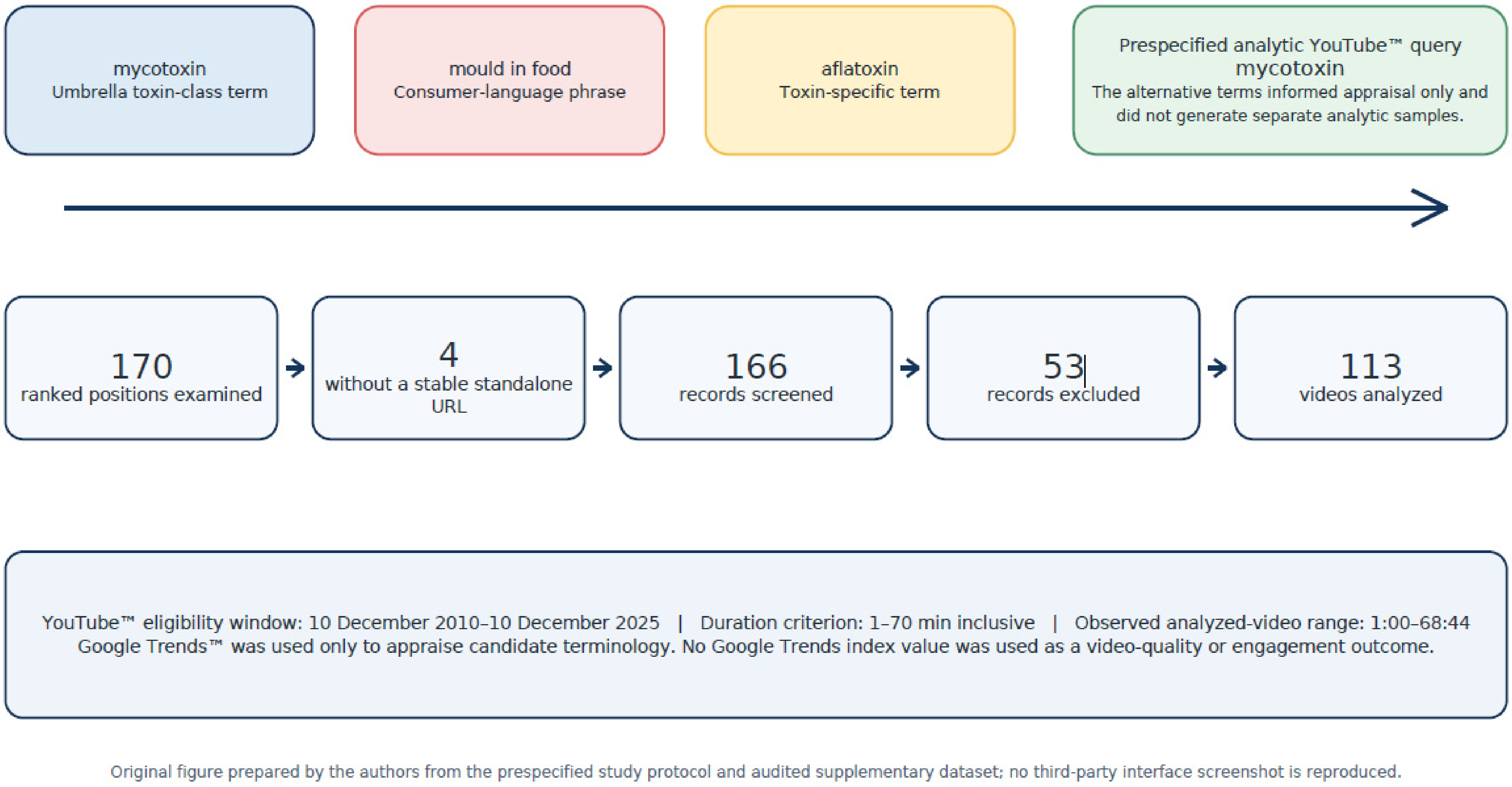

Figure S1. Google Trends–Supported Query Appraisal and Analytic YouTube Search Audit (Worldwide; all categories; Google Web Search; term-appraisal window: 1 January 2015–10 December 2025; appraisal date: 10 December 2025)
