# Supplementary material for: Public Communication of Foodborne Mycotoxin Risks on YouTube™: A Cross-Sectional Assessment of Video Quality, Reliability, and Educational Value
Source: Toxins (Basel). 2026 Aug 14;18(8):347. doi: 10.3390/toxins18080347 (PMC13517528; doi:10.3390/toxins18080347)
Supplement: Supplementary file 1 [file toxins-18-00347-s001.zip › File S1 Original Dataset and Reproducible Analysis.pdf]

Supplementary Materials: Public Communication of Foodborne Mycotoxin Risks on YouTube™: A Cross-Sectional Assessment of Video Quality, Reliability, and Educational Value

Ömer Faruk Yeşil and Ahmet Çelik

Supplementary File S1 — Original Dataset and Reproducible Analysis

**Study title**	Public Communication of Foodborne Mycotoxin Risks on YouTube™: A Cross-Sectional Assessment of Video Quality, Reliability, and Educational Value
**Search date**	10 December 2025
**Final included sample**	113
**Ranked search positions examined**	170
**Screened records**	166
**Excluded records**	53
**Scoring**	Two independent food-safety experts; rater-specific VCQ item scores and raw totals are retained.
**Analysis score**	VCQ, GQS, and modified DISCERN analyses use the arithmetic mean of the two independent rater totals.
**Derived fields**	Days since upload, rater means, interaction rate (%), and viewing rate (views/day) are formula-driven.
**Interaction rate**	((Likes + Comments) / Views) × 100
**Viewing rate**	Views / Days_Since_Upload
**VCQ item integrity**	For both raters, each 10-item VCQ sum matches the corresponding video-level VCQ total for all 113 videos.
**Workbook structure**	Raw/record-level data are separated from methods and results sheets. S6 reports item-level summaries; S7 preserves the item-level data and audit checks.
**Copyright**	All tables, calculations, and the VCQ chart in this workbook were created for this study and contain no third-party copyrighted

Record_ID	Video_SN	URL	Source_Type	Country	Continent	Views	Likes	Comments	Upload_Date	Days_Since_Upload	Duration_As_Recorded	VCQ_Rater_1	VCQ_Rater_2	VCQ_Mean	GQS_Rater_1	GQS_Rater_2	GQS_Mean	Modified_DISCERN_Rater_1	Modified_DISCERN_Rater_2	Modified_DISCERN_Mean	Interaction_Rate_Pct	Viewing_Rate_Views_Per_Day	Duration_Minutes
74	112	https://www.youtube.com/watch?v=m3CH3I26w5g&pp=ygUJbXljb3RveGlu	Company/commercial	Italy	Europe	119	2	0	2021-05-20	1665	1:00	15,00	15,00	15,00	4,00	4,00	4,00	5,00	5,00	5,00	1,681	0,071	1,00
73	149	https://www.youtube.com/watch?v=Gdelges1i-8&pp=ygUJbXljb3RveGlu	Company/commercial	Switzerland	Europe	695	7	0	2022-02-18	1391	1:01	9,00	9,00	9,00	3,00	3,00	3,00	4,00	4,00	4,00	1,007	0,500	1,02
65	137	https://www.youtube.com/watch?v=M4d5iuDfMFI&pp=ygUJbXljb3RveGlu	Company/commercial	India	Asia-Pacific	27	1	0	2024-03-27	623	1:08	15,00	15,00	15,00	4,00	4,00	4,00	4,00	4,00	4,00	3,704	0,043	1,13
77	32	https://www.youtube.com/watch?v=Z33ts-Hx3QE&pp=ygUJbXljb3RveGlu	Company/commercial	UK	Europe	1.935	5	0	2019-03-19	2458	1:26	17,00	17,00	17,00	5,00	5,00	5,00	5,00	5,00	5,00	0,258	0,787	1,43
56	40	https://www.youtube.com/watch?v=Jgu0jlET2hg&pp=ygUJbXljb3RveGlu	Company/commercial	China	Asia-Pacific	37	0	0	2025-03-31	254	1:39	18,00	18,00	18,00	5,00	5,00	5,00	5,00	5,00	5,00	0,000	0,146	1,65
9	58	https://www.youtube.com/watch?v=ayY9cwsT0uo&pp=ygUJbXljb3RveGlu	Academic/noncommercial	USA	North America	602	29	2	2020-05-19	2031	10:03	12,00	10,00	11,00	4,00	4,00	4,00	4,00	4,00	4,00	5,150	0,296	10,05
84	31	https://www.youtube.com/watch?v=zcogFjmAZjY&pp=ygUJbXljb3RveGlu	Company/commercial	USA	North America	169	0	0	2021-04-28	1687	10:53	4,00	4,00	4,00	2,00	2,00	2,00	3,00	3,00	3,00	0,000	0,100	10,88
22	118	https://www.youtube.com/watch?v=rLXzftZ1oyU&pp=ygUJbXljb3RveGlu	Academic/noncommercial	USA	North America	69	0	0	2025-04-30	224	11:03	10,00	10,00	10,00	3,00	3,00	3,00	3,00	3,00	3,00	0,000	0,308	11,05
29	80	https://www.youtube.com/watch?v=yX5KCHlEymU&pp=ygUJbXljb3RveGlu	Academic/noncommercial	USA	North America	854	17	0	2022-03-21	1360	11:41	15,00	15,00	15,00	4,00	4,00	4,00	4,00	4,00	4,00	1,991	0,628	11,68
66	158	https://www.youtube.com/watch?v=8wqXRa91oHs&pp=ygUJbXljb3RveGlu	Company/commercial	India	Asia-Pacific	11	0	0	2024-03-08	642	11:51	18,00	18,00	18,00	5,00	5,00	5,00	5,00	5,00	5,00	0,000	0,017	11,85
72	124	https://www.youtube.com/watch?v=ypV4fV3nldw&pp=ygUJbXljb3RveGlu	Company/commercial	Switzerland	Europe	86	0	0	2025-04-15	239	11:51	16,00	15,00	15,50	5,00	5,00	5,00	5,00	4,00	4,50	0,000	0,360	11,85
10	60	https://www.youtube.com/watch?v=oVZt5fm8u8Y&pp=ygUJbXljb3RveGlu	Academic/noncommercial	USA	North America	10.912	237	12	2019-10-02	2261	13:05	8,00	8,00	8,00	3,00	3,00	3,00	4,00	3,00	3,50	2,282	4,826	13,08
98	134	https://www.youtube.com/watch?v=JqLEPXYO6zA&pp=ygUJbXljb3RveGlu	Company/commercial	USA	North America	15	0	0	2025-01-22	322	13:16	15,00	15,00	15,00	3,00	3,00	3,00	4,00	4,00	4,00	0,000	0,047	13,27
51	75	https://www.youtube.com/watch?v=YDDr_Z0j0Y0&pp=ygUJbXljb3RveGlu0gcJCccJAYcqIYzv	Company/commercial	Austria	Europe	739	8	0	2013-09-17	4467	13:44	16,00	14,00	15,00	5,00	5,00	5,00	5,00	5,00	5,00	1,083	0,165	13,73
58	103	https://www.youtube.com/watch?v=UiF2RrD0p_E&pp=ygUJbXljb3RveGlu	Company/commercial	China	Asia-Pacific	29	0	0	2021-05-28	1657	13:44	8,00	8,00	8,00	3,00	3,00	3,00	3,00	3,00	3,00	0,000	0,018	13,73
60	141	https://www.youtube.com/watch?v=y3U4iqUBhPY&pp=ygUJbXljb3RveGlu	Company/commercial	China	Asia-Pacific	270	9	0	2020-07-28	1961	14:15	18,00	18,00	18,00	5,00	5,00	5,00	5,00	5,00	5,00	3,333	0,138	14,25
5	147	https://www.youtube.com/watch?v=eRqlzPGpPGA&pp=ygUJbXljb3RveGlu	Academic/noncommercial	UK	Europe	1.100	38	0	2025-02-03	310	14:54	7,00	7,00	7,00	4,00	4,00	4,00	2,00	2,00	2,00	3,455	3,548	14,90
13	90	https://www.youtube.com/watch?v=-UVeD7 k6EU&pp=ygUJbXljb3RveGlu	Academic/noncommercial	USA	North America	235	10	1	2024-01-25	685	15:29	10,00	8,00	9,00	4,00	4,00	4,00	4,00	4,00	4,00	4,681	0,343	15,48
108	167	https://www.youtube.com/watch?v=W1MF4LBe_PY&pp=ygUJbXljb3RveGlu	Company/commercial	USA	North America	1.100	2	1	2025-06-19	174	15:52	18,00	16,00	17,00	5,00	5,00	5,00	5,00	5,00	5,00	0,273	6,322	15,87
110	170	https://www.youtube.com/watch?v=W3hHfup5Cuo&pp=ygUJbXljb3RveGlu	Company/commercial	USA	North America	121	0	0	2024-01-16	694	16:06	15,00	15,00	15,00	4,00	4,00	4,00	4,00	4,00	4,00	0,000	0,174	16,10
92	86	https://www.youtube.com/watch?v=C67570-K3o&pp=ygUJbXljb3RveGlu	Company/commercial	USA	North America	857	4	1	2017-12-20	2912	16:26	11,00	11,00	11,00	3,00	3,00	3,00	3,00	3,00	3,00	0,583	0,294	16,43
2	46	https://www.youtube.com/watch?v=vwXsJaFjbXg&pp=ygUJbXljb3RveGlu	Academic/noncommercial	Australia	Asia-Pacific	4.440	292	84	2025-07-01	162	17:01	10,00	9,00	9,50	4,00	3,00	3,50	2,00	2,00	2,00	8,468	27,407	17,02
104	155	https://www.youtube.com/watch?v=5ZYY_X3swYc&pp=ygUJbXljb3RveGlu	Company/commercial	USA	North America	45	1	0	2021-01-30	1775	17:11	12,00	12,00	12,00	4,00	4,00	4,00	4,00	4,00	4,00	2,222	0,025	17,18
7	34	https://www.youtube.com/watch?v=LV5pQ6dBvDg&pp=ygUJbXljb3RveGlu	Academic/noncommercial	USA	North America	2.668	94	15	2025-02-06	307	17:14	7,00	7,00	7,00	3,00	3,00	3,00	3,00	3,00	3,00	4,085	8,691	17,23
6	110	https://www.youtube.com/watch?v=kuxSTQrilxg&pp=ygUJbXljb3RveGlu	Academic/noncommercial	UK	Europe	1.699	27	1	2015-03-26	3912	18:53	8,00	7,00	7,50	4,00	3,00	3,50	2,00	2,00	2,00	1,648	0,434	18,88
19	98	https://www.youtube.com/watch?v=AXESIFTjIJA&pp=ygUJbXljb3RveGlu	Academic/noncommercial	USA	North America	430	13	0	2024-08-20	477	19:12	7,00	6,00	6,50	4,00	4,00	4,00	4,00	3,00	3,50	3,023	0,901	19,20
102	148	https://www.youtube.com/watch?v=CXqAiWfNvL0&pp=ygUJbXljb3RveGlu	Company/commercial	USA	North America	145	0	0	2023-12-15	726	19:37	17,00	17,00	17,00	4,00	4,00	4,00	4,00	4,00	4,00	0,000	0,200	19,62
49	38	https://www.youtube.com/watch?v=0NSkgKWQcRQ&pp=ygUJbXljb3RveGlu	Company/commercial	Austria	Europe	122	2	0	2020-04-02	2078	2:08	14,00	14,00	14,00	4,00	4,00	4,00	4,00	4,00	4,00	1,639	0,059	2,13
46	18	https://www.youtube.com/watch?v=MXL1HR7Ry34&pp=ygUJbXljb3RveGlu	Company/commercial	Austria	Europe	810	14	0	2020-10-09	1888	2:15	20,00	20,00	20,00	5,00	5,00	5,00	5,00	5,00	5,00	1,728	0,429	2,25
44	13	https://www.youtube.com/watch?v=06dRcpISfso&pp=ygUJbXljb3RveGlu	Company/commercial	Austria	Europe	16.455	262	7	2020-10-29	1868	2:19	17,00	17,00	17,00	4,00	4,00	4,00	5,00	5,00	5,00	1,635	8,809	2,32
61	63	https://www.youtube.com/watch?v=y7Xg-QMSn8E&pp=ygUJbXljb3RveGlu	Company/commercial	Denmark	Europe	692	10	0	2021-03-08	1738	2:20	14,00	14,00	14,00	4,00	4,00	4,00	4,00	4,00	4,00	1,445	0,398	2,33
55	39	https://www.youtube.com/watch?v=DUKZ4fgQMds&pp=ygUJbXljb3RveGlu	Company/commercial	Belgium	Europe	823	8	2	2023-05-28	927	2:25	16,00	16,00	16,00	4,00	4,00	4,00	5,00	4,00	4,50	1,215	0,888	2,42
43	12	https://www.youtube.com/watch?v=2RXPzigEVLY&pp=ygUJbXljb3RveGlu	Company/commercial	Austria	Europe	343	7	0	2020-02-20	2120	2:30	9,00	9,00	9,00	3,00	3,00	3,00	4,00	3,00	3,50	2,041	0,162	2,50
48	23	https://www.youtube.com/watch?v=6EfYwtlctkk&pp=ygUJbXljb3RveGlu	Company/commercial	Austria	Europe	16.318	198	5	2018-11-05	2592	2:30	18,00	18,00	18,00	5,00	5,00	5,00	5,00	5,00	5,00	1,244	6,296	2,50
38	24	https://www.youtube.com/watch?v=h7mldtGDfNU&pp=ygUJbXljb3RveGlu	Company/commercial	Germany	Europe	1.753	6	0	2018-02-14	2856	2:40	11,00	11,00	11,00	4,00	4,00	4,00	2,00	2,00	2,00	0,342	0,614	2,67
82	22	https://www.youtube.com/watch?v=wVEXmi8ZVxo&pp=ygUJbXljb3RveGlu	Company/commercial	USA	North America	1.536	14	0	2018-06-24	2726	2:40	12,00	12,00	12,00	4,00	4,00	4,00	4,00	4,00	4,00	0,911	0,563	2,67
40	72	https://www.youtube.com/watch?v=NADUmlpbI9w&pp=ygUJbXljb3RveGlu	Company/commercial	Australia	Asia-Pacific	1.030	16	2	2022-05-10	1310	2:46	4,00	4,00	4,00	2,00	2,00	2,00	2,00	2,00	2,00	1,748	0,786	2,77
88	53	https://www.youtube.com/watch?v=BVisg-URJik&pp=ygUJbXljb3RveGlu	Company/commercial	USA	North America	204	2	1	2020-09-08	1919	2:55	14,00	14,00	14,00	4,00	4,00	4,00	4,00	4,00	4,00	1,471	0,106	2,92
62	108	https://www.youtube.com/watch?v=1WDR3fx5CBc&pp=ygUJbXljb3RveGlu	Company/commercial	France	Europe	72	2	0	2022-10-10	1157	20:00	16,00	16,00	16,00	4,00	4,00	4,00	5,00	5,00	5,00	2,778	0,062	20,00
59	127	https://www.youtube.com/watch?v=izmQt5GDGOc&pp=ygUJbXljb3RveGlu	Company/commercial	China	Asia-Pacific	31	1	1	2024-09-25	441	22:13	18,00	18,00	18,00	4,00	4,00	4,00	5,00	5,00	5,00	6,452	0,070	22,22
34	150	https://www.youtube.com/watch?v=XdiF9JbbYaY&pp=ygUJbXljb3RveGlu	Academic/noncommercial	USA	North America	28	2	0	2025-05-07	217	23:42	11,00	11,00	11,00	3,00	3,00	3,00	2,00	2,00	2,00	7,143	0,129	23,70
47	19	https://www.youtube.com/watch?v=3ejOFH2vkNo&pp=ygUJbXljb3RveGlu0gcJCccJAYcqIYzv	Company/commercial	Austria	Europe	2.641	19	0	2013-09-17	4467	24:25	18,00	18,00	18,00	5,00	4,00	4,50	5,00	5,00	5,00	0,719	0,591	24,42
100	143	https://www.youtube.com/watch?v=IfVNO4Hw2Lw&pp=ygUJbXljb3RveGlu	Company/commercial	USA	North America	133	1	0	2024-08-05	492	24:49	1,00	1,00	1,00	1,00	1,00	1,00	0,00	0,00	0,00	0,752	0,270	24,82
28	130	https://www.youtube.com/watch?v=e6PiIBXNVEI&pp=ygUJbXljb3RveGlu0gcJCccJAYcqIYzv	Academic/noncommercial	USA	North America	21	0	0	2025-07-01	162	26:29	3,00	3,00	3,00	3,00	3,00	3,00	2,00	2,00	2,00	0,000	0,130	26,48
83	30	https://www.youtube.com/watch?v=mtyjk0MDyAQ&pp=ygUJbXljb3RveGlu	Company/commercial	USA	North America	259	1	0	2023-10-17	785	3:00	12,00	12,00	12,00	4,00	4,00	4,00	4,00	4,00	4,00	0,386	0,330	3,00
45	15	https://www.youtube.com/watch?v=lwC1VvSk9AY&pp=ygUJbXljb3RveGlu	Company/commercial	Austria	Europe	37.172	255	6	2018-03-19	2823	3:07	11,00	11,00	11,00	4,00	4,00	4,00	5,00	5,00	5,00	0,702	13,168	3,12
11	66	https://www.youtube.com/watch?v=06yJ9xDabFM&pp=ygUJbXljb3RveGlu	Academic/noncommercial	USA	North America	156.108	6.200	423	2017-10-10	2983	3:10	8,00	8,00	8,00	4,00	4,00	4,00	4,00	4,00	4,00	4,243	52,333	3,17
78	42	https://www.youtube.com/watch?v=uo6yeOFdAcQ&pp=ygUJbXljb3RveGlu	Company/commercial	UK	Europe	748	24	2	2022-03-21	1360	3:11	17,00	19,00	18,00	4,00	4,00	4,00	5,00	5,00	5,00	3,476	0,550	3,18
57	52	https://www.youtube.com/watch?v=SXZDNAfCYb0&pp=ygUJbXljb3RveGlu	Company/commercial	China	Asia-Pacific	272	4	0	2024-06-05	553	3:14	17,00	17,00	17,00	5,00	4,00	4,50	5,00	5,00	5,00	1,471	0,492	3,23
81	10	https://www.youtube.com/watch?v=rC00XwKqm9c&pp=ygUJbXljb3RveGlu	Company/commercial	USA	North America	1.688	26	5	2020-04-10	2070	3:30	8,00	8,00	8,00	3,00	3,00	3,00	3,00	4,00	3,50	1,836	0,815	3,50
97	133	https://www.youtube.com/watch?v=6IKc-aJwyzQ&pp=ygUJbXljb3RveGlu	Company/commercial	USA	North America	79	6	0	2025-05-20	204	3:35	12,00	12,00	12,00	4,00	4,00	4,00	4,00	4,00	4,00	7,595	0,387	3,58
90	67	https://www.youtube.com/watch?v=LjsrD3eO55c&pp=ygUJbXljb3RveGlu	Company/commercial	USA	North America	1.195	8	0	2017-11-16	2946	3:37	10,00	10,00	10,00	3,00	3,00	3,00	4,00	4,00	4,00	0,669	0,406	3,62
31	114	https://www.youtube.com/watch?v=QHOY9SbQsj0&pp=ygUJbXljb3RveGlu	Academic/noncommercial	USA	North America	54	5	2	2025-06-04	189	3:39	6,00	6,00	6,00	3,00	3,00	3,00	2,00	2,00	2,00	12,963	0,286	3,65
75	5	https://www.youtube.com/watch?v=e5Lwa2kVvhE&pp=ygUJbXljb3RveGlu	Company/commercial	Japan	Asia-Pacific	7.414	67	2	2021-10-30	1502	3:42	18,00	18,00	18,00	5,00	5,00	5,00	5,00	5,00	5,00	0,931	4,936	3,70
63	21	https://www.youtube.com/watch?v=4mSr5NN9YS0&pp=ygUJbXljb3RveGlu	Company/commercial	India	Asia-Pacific	627	13	0	2023-09-04	828	3:55	9,00	9,00	9,00	3,00	3,00	3,00	3,00	3,00	3,00	2,073	0,757	3,92
89	64	https://www.youtube.com/watch?v=syRJvupPMW8&pp=ygUJbXljb3RveGlu	Company/commercial	USA	North America	122	4	0	2025-03-05	280	3:57	15,00	15,00	15,00	4,00	3,00	3,50	4,00	4,00	4,00	3,279	0,436	3,95
54	169	https://www.youtube.com/watch?v=iyrRxQjiiZM&pp=ygUJbXljb3RveGlu	Company/commercial	Austria	Europe	913	7	1	2017-01-03	3263	30:06	16,00	16,00	16,00	4,00	4,00	4,00	5,00	5,00	5,00	0,876	0,280	30,10

87	47	https://www.youtube.com/watch?v=mJzqGk8qFJs&pp=ygUJbXljb3RveGlu	Company/commercial	USA	North America	402	17	1	2018-03-28	2814	30:12	12,00	12,00	12,00	4,00	4,00	4,00	4,00	4,00	4,00	4,478	0,143	30,20
16	93	https://www.youtube.com/watch?v=fANOxBu-lFI&pp=ygUJbXljb3RveGlu	Academic/noncommercial	USA	North America	2.819	3	0	2025-04-22	232	31:32	12,00	12,00	12,00	4,00	4,00	4,00	3,00	3,00	3,00	0,106	12,151	31,53
103	152	https://www.youtube.com/watch?v=Mn0r20ui9I0&pp=ygUJbXljb3RveGlu0gcJCccJAYcqIYzv	Company/commercial	USA	North America	84	3	0	2021-07-01	1623	31:41	12,00	12,00	12,00	4,00	3,00	3,50	4,00	4,00	4,00	3,571	0,052	31,68

69	55	https://www.youtube.com/watch?v=pX_0xkAdVr0&pp=ygUJbXljb3RveGlu	Company/commercial	Ireland	Europe	411	2	1	2021-02-01	1773	32:48	15,00	15,00	15,00	5,00	5,00	5,00	5,00	5,00	5,00	0,730	0,232	32,80
4	94	https://www.youtube.com/watch?v=wOHjRoxgg_g&pp=ygUJbXljb3RveGlu	Academic/noncommercial	Canada	North America	1.423	9	0	2024-04-24	595	33:07	12,00	12,00	12,00	4,00	4,00	4,00	2,00	3,00	2,50	0,632	2,392	33,12
30	102	https://www.youtube.com/watch?v=g05gZga6bbs&pp=ygUJbXljb3RveGlu	Academic/noncommercial	USA	North America	19.808	876	173	2021-06-07	1647	34:55	11,00	11,00	11,00	3,00	3,00	3,00	2,00	2,00	2,00	5,296	12,027	34,92
18	96	https://www.youtube.com/watch?v=I35UWdz65_Y&t=482s&pp=ygUJbXljb3RveGlu	Academic/noncommercial	USA	North America	3.664	135	21	2024-09-02	464	36:18	5,00	5,00	5,00	4,00	4,00	4,00	3,00	3,00	3,00	4,258	7,897	36,30
36	156	https://www.youtube.com/watch?v=TTu58R2J5I0&pp=ygUJbXljb3RveGlu	Academic/noncommercial	Australia	Asia-Pacific	107	6	0	2023-10-22	780	36:34	16,00	16,00	16,00	4,00	4,00	4,00	3,00	3,00	3,00	5,607	0,137	36,57
42	139	https://www.youtube.com/watch?v=OZbzQN4DnfI&pp=ygUJbXljb3RveGlu	Company/commercial	Australia	Asia-Pacific	82	0	0	2020-12-01	1835	38:50	19,00	19,00	19,00	5,00	5,00	5,00	5,00	5,00	5,00	0,000	0,045	38,83
3	157	https://www.youtube.com/watch?v=-Y-dIZwgCtg&pp=ygUJbXljb3RveGlu	Academic/noncommercial	India	Asia-Pacific	43	5	0	2022-06-21	1268	39:18	16,00	14,00	15,00	5,00	5,00	5,00	3,00	3,00	3,00	11,628	0,034	39,30
96	117	https://www.youtube.com/watch?v=k-oW5lEGqdg&pp=ygUJbXljb3RveGlu	Company/commercial	USA	North America	137	1	0	2022-11-09	1127	39:44	14,00	14,00	14,00	4,00	4,00	4,00	4,00	4,00	4,00	0,730	0,122	39,73
71	115	https://www.youtube.com/watch?v=HUI8nBjkSCA&pp=ygUJbXljb3RveGlu	Company/commercial	Switzerland	Europe	90	0	0	2025-02-21	292	4:07	16,00	15,00	15,50	4,00	3,00	3,50	4,00	4,00	4,00	0,000	0,308	4,12
79	4	https://www.youtube.com/watch?v=7IcPPUTi7Y4&pp=ygUJbXljb3RveGlu	Company/commercial	USA	North America	2.448	8	0	2017-07-28	3057	4:11	16,00	15,00	15,50	5,00	5,00	5,00	5,00	5,00	5,00	0,327	0,801	4,18
17	95	https://www.youtube.com/watch?v=FXej_lmBS54&pp=ygUJbXljb3RveGlu0gcJCccJAYcqIYzv	Academic/noncommercial	USA	North America	56	3	0	2025-06-12	181	4:14	9,00	9,00	9,00	4,00	4,00	4,00	3,00	3,00	3,00	5,357	0,309	4,23
26	154	https://www.youtube.com/watch?v=-68pKozZPmU&pp=ygUJbXljb3RveGlu	Academic/noncommercial	USA	North America	81	0	0	2011-09-08	5207	4:19	10,00	10,00	10,00	3,00	3,00	3,00	3,00	3,00	3,00	0,000	0,016	4,32
85	33	https://www.youtube.com/watch?v=ipg21vcS_iU&pp=ygUJbXljb3RveGlu	Company/commercial	USA	North America	845	8	1	2020-05-06	2044	4:22	4,00	4,00	4,00	2,00	2,00	2,00	3,00	3,00	3,00	1,065	0,413	4,37
68	145	https://www.youtube.com/watch?v=YbT7RseXPd0&pp=ygUJbXljb3RveGlu	Company/commercial	Netherlands	Europe	63	6	1	2024-12-04	371	4:24	18,00	18,00	18,00	5,00	5,00	5,00	5,00	5,00	5,00	11,111	0,170	4,40
41	82	https://www.youtube.com/watch?v=nMrvA39Xcck&pp=ygUJbXljb3RveGlu	Company/commercial	Australia	Asia-Pacific	48	5	0	2025-03-03	282	4:27	4,00	4,00	4,00	2,00	2,00	2,00	2,00	2,00	2,00	10,417	0,170	4,45
80	6	https://www.youtube.com/watch?v=83PHhf4fOuI&pp=ygUJbXljb3RveGlu	Company/commercial	USA	North America	484	14	4	2021-04-14	1701	4:28	17,00	17,00	17,00	5,00	5,00	5,00	5,00	5,00	5,00	3,719	0,285	4,47
101	146	https://www.youtube.com/watch?v=lD8SdNCMMfE&pp=ygUJbXljb3RveGlu0gcJCccJAYcqIYzv	Company/commercial	USA	North America	25	3	1	2025-04-18	236	4:29	12,00	11,00	11,50	4,00	4,00	4,00	4,00	4,00	4,00	16,000	0,106	4,48
105	159	https://www.youtube.com/watch?v=j_4qQfnD9II&pp=ygUJbXljb3RveGlu	Company/commercial	USA	North America	1.200	54	9	2023-11-29	742	40:10	12,00	12,00	12,00	4,00	4,00	4,00	4,00	4,00	4,00	5,250	1,617	40,17
32	125	https://www.youtube.com/watch?v=sc-2Zu4kM7w&pp=ygUJbXljb3RveGlu	Academic/noncommercial	USA	North America	25	2	0	2025-04-04	250	41:01	6,00	6,00	6,00	3,00	3,00	3,00	2,00	2,00	2,00	8,000	0,100	41,02
15	92	https://www.youtube.com/watch?v=eLPPc3wF1rY&pp=ygUJbXljb3RveGlu	Academic/noncommercial	USA	North America	457	22	3	2025-04-07	247	42:36	5,00	5,00	5,00	4,00	4,00	4,00	3,00	3,00	3,00	5,470	1,850	42,60
53	121	https://www.youtube.com/watch?v=yfZOtrzCbs8&pp=ygUJbXljb3RveGlu	Company/commercial	Austria	Europe	864	3	2	2019-02-26	2479	45:41	11,00	11,00	11,00	4,00	4,00	4,00	5,00	5,00	5,00	0,579	0,349	45,68
95	99	https://www.youtube.com/watch?v=G8_pM_yqaSE&pp=ygUJbXljb3RveGlu	Company/commercial	USA	North America	6.802	221	51	2023-10-19	783	46:54	12,00	12,00	12,00	4,00	4,00	4,00	4,00	4,00	4,00	3,999	8,687	46,90
50	70	https://www.youtube.com/watch?v=CdnH11eqsB4&pp=ygUJbXljb3RveGlu	Company/commercial	Austria	Europe	304	6	7	2021-03-11	1735	47:53	16,00	16,00	16,00	4,00	4,00	4,00	3,00	3,00	3,00	4,276	0,175	47,88
52	104	https://www.youtube.com/watch?v=Wru21Twj6jM&pp=ygUJbXljb3RveGlu	Company/commercial	Austria	Europe	191	3	1	2021-03-11	1735	47:54	11,00	11,00	11,00	4,00	4,00	4,00	4,00	4,00	4,00	2,094	0,110	47,90
93	89	https://www.youtube.com/watch?v=ZCauPu_kWKo&pp=ygUJbXljb3RveGlu	Company/commercial	USA	North America	210	1	1	2021-11-03	1498	48:48	3,00	3,00	3,00	2,00	2,00	2,00	2,00	2,00	2,00	0,952	0,140	48,80
70	78	https://www.youtube.com/watch?v=L1319tg5ct4&pp=ygUJbXljb3RveGlu	Company/commercial	Ireland	Europe	454	10	0	2020-07-24	1965	49:03	9,00	9,00	9,00	3,00	3,00	3,00	4,00	4,00	4,00	2,203	0,231	49,05
20	101	https://www.youtube.com/watch?v=aXjMVrYp1fg&pp=ygUJbXljb3RveGlu	Academic/noncommercial	USA	North America	29	0	0	2025-01-22	322	5:03	4,00	4,00	4,00	3,00	3,00	3,00	3,00	3,00	3,00	0,000	0,090	5,05
1	36	https://www.youtube.com/watch?v=t-7YMrPnDuA&pp=ygUJbXljb3RveGlu	Academic/noncommercial	Germany	Europe	3.237	5	0	2011-06-09	5298	5:23	6,00	6,00	6,00	4,00	4,00	4,00	3,00	3,00	3,00	0,154	0,611	5,38
8	49	https://www.youtube.com/watch?v=_7Nj1JOp2ik&pp=ygUJbXljb3RveGlu	Academic/noncommercial	USA	North America	73.028	2.400	329	2023-08-02	861	5:29	13,00	13,00	13,00	4,00	4,00	4,00	3,00	3,00	3,00	3,737	84,818	5,48
33	142	https://www.youtube.com/watch?v=tkbOwH7fss8&pp=ygUJbXljb3RveGlu	Academic/noncommercial	USA	North America	392	1	0	2011-07-08	5269	5:29	16,00	16,00	16,00	5,00	5,00	5,00	5,00	5,00	5,00	0,255	0,074	5,48
99	140	https://www.youtube.com/watch?v=nD_lxUjamXg&pp=ygUJbXljb3RveGlu	Company/commercial	USA	North America	206	0	0	2022-03-10	1371	5:30	16,00	16,00	16,00	4,00	4,00	4,00	4,00	4,00	4,00	0,000	0,150	5,50
25	65	https://www.youtube.com/watch?v=Vrd7YUjR81M&pp=ygUJbXljb3RveGlu	Academic/noncommercial	USA	North America	346	2	0	2015-10-06	3718	5:57	15,00	15,00	15,00	4,00	4,00	4,00	3,00	3,00	3,00	0,578	0,093	5,95
67	105	https://www.youtube.com/watch?v=LWV03__a_fA&pp=ygUJbXljb3RveGlu	Company/commercial	Netherlands	Europe	1.076	15	0	2022-02-18	1391	51:10	18,00	18,00	18,00	5,00	5,00	5,00	5,00	5,00	5,00	1,394	0,774	51,17
35	151	https://www.youtube.com/watch?v=CuBMBwb0uMk&pp=ygUJbXljb3RveGlu	Academic/noncommercial	USA	North America	18	0	0	2024-08-27	470	52:29	11,00	11,00	11,00	3,00	3,00	3,00	3,00	3,00	3,00	0,000	0,038	52,48
24	165	https://www.youtube.com/watch?v=5IjFP23BRlM&pp=ygUJbXljb3RveGlu0gcJCccJAYcqIYzv	Academic/noncommercial	USA	North America	145	6	0	2025-02-07	306	54:08	12,00	12,00	12,00	3,00	3,00	3,00	3,00	3,00	3,00	4,138	0,474	54,13
27	76	https://www.youtube.com/watch?v=lcsas5zCma4&pp=ygUJbXljb3RveGlu	Academic/noncommercial	USA	North America	5.650	131	32	2019-02-20	2485	54:58	8,00	8,00	8,00	3,00	4,00	3,50	3,00	4,00	3,50	2,885	2,274	54,97
109	168	https://www.youtube.com/watch?v=O7QvVTAAXNA&pp=ygUJbXljb3RveGlu	Company/commercial	USA	North America	113	2	0	2021-05-31	1654	55:22	12,00	12,00	12,00	4,00	4,00	4,00	4,00	4,00	4,00	1,770	0,068	55,37
12	79	https://www.youtube.com/watch?v=pk_HiEQnsoY&pp=ygUJbXljb3RveGlu	Academic/noncommercial	USA	North America	6.839	120	21	2017-10-26	2967	55:29	5,00	5,00	5,00	3,00	3,00	3,00	3,00	3,00	3,00	2,062	2,305	55,48
94	97	https://www.youtube.com/watch?v=kP1Zy5tVJIY&pp=ygUJbXljb3RveGlu	Company/commercial	USA	North America	306	6	0	2021-05-31	1654	57:20	6,00	6,00	6,00	2,00	2,00	2,00	2,00	2,00	2,00	1,961	0,185	57,33
106	161	https://www.youtube.com/watch?v=RxNAuk5k_Jg&pp=ygUJbXljb3RveGlu	Company/commercial	USA	North America	1.300	376	33	2018-07-05	2715	57:45	12,00	12,00	12,00	4,00	4,00	4,00	4,00	4,00	4,00	31,462	0,479	57,75
113	106	https://www.youtube.com/watch?v=jLagXsvp1fo&pp=ygUJbXljb3RveGlu	Company/commercial	Greece	Europe	140	1	0	2023-07-17	877	59:23	12,00	13,00	12,50	4,00	4,00	4,00	4,00	4,00	4,00	0,714	0,160	59,38
37	9	https://www.youtube.com/watch?v=2obpRxhz6qU&pp=ygUJbXljb3RveGlu	Company/commercial	Germany	Europe	6.218	26	0	2022-03-18	1363	6:19	8,00	8,00	8,00	3,00	3,00	3,00	2,00	2,00	2,00	0,418	4,562	6,32
23	160	https://www.youtube.com/watch?v=UPxwZCo7ZoI&pp=ygUJbXljb3RveGlu	Academic/noncommercial	USA	North America	65	2	0	2024-11-01	404	6:26	15,00	15,00	15,00	4,00	4,00	4,00	3,00	3,00	3,00	3,077	0,161	6,43
64	56	https://www.youtube.com/watch?v=rrhsl-sNrus&pp=ygUJbXljb3RveGlu	Company/commercial	India	Asia-Pacific	1.021	32	2	2022-02-23	1386	6:40	16,00	16,00	16,00	5,00	5,00	5,00	5,00	5,00	5,00	3,330	0,737	6,67
91	73	https://www.youtube.com/watch?v=qcRBK0KO1XE&pp=ygUJbXljb3RveGlu	Company/commercial	USA	North America	10.107	141	8	2023-05-05	950	68:44	15,00	15,00	15,00	4,00	4,00	4,00	5,00	5,00	5,00	1,474	10,639	68,73
112	1	https://www.youtube.com/watch?v=7Dm7neAxoqQ&t=12s&pp=ygUJbXljb3RveGlu	Company/commercial	Greece	Europe	19.183	92	0	2020-04-15	2065	7:34	15,00	14,00	14,50	4,00	4,00	4,00	4,00	3,00	3,50	0,480	9,290	7,57
76	41	https://www.youtube.com/watch?v=2iX8CJUp_G8&pp=ygUJbXljb3RveGlu	Company/commercial	Serbia	Europe	59	2	0	2025-03-13	272	7:40	17,00	17,00	17,00	5,00	5,00	5,00	5,00	5,00	5,00	3,390	0,217	7,67
21	109	https://www.youtube.com/watch?v=34xpPos07Eo&pp=ygUJbXljb3RveGlu	Academic/noncommercial	USA	North America	285	10	5	2025-04-18	236	8:14	15,00	15,00	15,00	2,00	2,00	2,00	3,00	3,00	3,00	5,263	1,208	8,23
39	129	https://www.youtube.com/watch?v=FpheRy9f9zE&pp=ygUJbXljb3RveGlu	Company/commercial	Germany	Europe	52	1	0	2024-03-13	637	8:18	17,00	17,00	17,00	4,00	4,00	4,00	3,00	3,00	3,00	1,923	0,082	8,30
111	128	https://www.youtube.com/watch?v=dTMjPZkgnm4&pp=ygUJbXljb3RveGlu	Company/commercial	USA	North America	684	1	0	2017-12-17	2915	8:23	15,00	15,00	15,00	4,00	3,00	3,50	4,00	4,00	4,00	0,146	0,235	8,38
14	91	https://www.youtube.com/watch?v=HWyUy86D2L8&pp=ygUJbXljb3RveGlu	Academic/noncommercial	USA	North America	29.311	1.000	0	2022-02-09	1400	7:32	13,00	13,00	13,00	4,00	4,00	4,00	4,00	4,00	4,00	3,412	20,936	7,53
107	163	https://www.youtube.com/watch?v=EnOLzvjofFM&pp=ygUJbXljb3RveGlu	Company/commercial	USA	North America	243.785	6.000	374	2022-05-04	1316	9:45	10,00	10,00	10,00	3,00	3,00	3,00	2,00	2,00	2,00	2,615	185,247	9,75
86	45	https://www.youtube.com/watch?v=sT47MHnPc40&pp=ygUJbXljb3RveGlu	Company/commercial	USA	North America	6.404	29	0	2018-03-05	2837	9:54	5,00	5,00	5,00	2,00	2,00	2,00	4,00	4,00	4,00	0,453	2,257	9,90

Search_Rank	Canonical_URL	Screening_Status
1	https://www.youtube.com/watch?v=7Dm7neAxoqQ&t=12s&pp=ygUJbXljb3RveGlu	Excluded
2	https://www.youtube.com/watch?v=pzUi6gxuy3g&t=1s&pp=ygUJbXljb3RveGlu	Excluded
3	https://www.youtube.com/watch?v=cIhjQDQYzDs&pp=ygUJbXljb3RveGlu	Excluded
4	https://www.youtube.com/watch?v=7IcPPUTi7Y4&pp=ygUJbXljb3RveGlu	Included in analysis
5	https://www.youtube.com/watch?v=e5Lwa2kVvhE&pp=ygUJbXljb3RveGlu	Included in analysis
6	https://www.youtube.com/watch?v=83PHhf4fOuI&pp=ygUJbXljb3RveGlu	Included in analysis
7	https://www.youtube.com/watch?v=GMEmiH-Njmc&pp=ygUJbXljb3RveGlu	Excluded
8	https://www.youtube.com/watch?v=ONvvxPB25pE&pp=ygUJbXljb3RveGlu	Excluded
9	https://www.youtube.com/watch?v=2obpRxhz6qU&pp=ygUJbXljb3RveGlu	Included in analysis
10	https://www.youtube.com/watch?v=rC00XwKqm9c&pp=ygUJbXljb3RveGlu	Included in analysis
11	https://www.youtube.com/watch?v=AYe9k3TDxdo&pp=ygUJbXljb3RveGlu	Excluded
12	https://www.youtube.com/watch?v=2RXPzigEVLY&pp=ygUJbXljb3RveGlu	Included in analysis
13	https://www.youtube.com/watch?v=06dRcpISfso&pp=ygUJbXljb3RveGlu	Excluded
14	https://www.youtube.com/watch?v=xBUJ0SKLHKY&pp=ygUJbXljb3RveGlu	Excluded
15	https://www.youtube.com/watch?v=lwC1VvSk9AY&pp=ygUJbXljb3RveGlu	Included in analysis
16	https://www.youtube.com/watch?v=GkCPXu137qI&pp=ygUJbXljb3RveGlu	Excluded
17	https://www.youtube.com/watch?v=K3i0pEEd8EQ&pp=ygUJbXljb3RveGlu	Excluded
18	https://www.youtube.com/watch?v=MXL1HR7Ry34&pp=ygUJbXljb3RveGlu	Included in analysis
19	https://www.youtube.com/watch?v=3ejOFH2vkNo&pp=ygUJbXljb3RveGlu0gcJCccJAYcqIYzv	Excluded
20	https://www.youtube.com/watch?v=yRqrcoNNnsI&pp=ygUJbXljb3RveGlu	Excluded
21	https://www.youtube.com/watch?v=4mSr5NN9YS0&pp=ygUJbXljb3RveGlu	Included in analysis
22	https://www.youtube.com/watch?v=wVEXmi8ZVxo&pp=ygUJbXljb3RveGlu	Included in analysis
23	https://www.youtube.com/watch?v=6EfYwtlctkk&pp=ygUJbXljb3RveGlu	Included in analysis
24	https://www.youtube.com/watch?v=h7mldtGDfNU&pp=ygUJbXljb3RveGlu	Included in analysis
25	https://www.youtube.com/watch?v=IubXH9gFZD4&pp=ygUJbXljb3RveGlu	Excluded
26	https://www.youtube.com/watch?v=cZ7yxTXmBvU&pp=ygUJbXljb3RveGlu	Included in analysis
27	https://www.youtube.com/watch?v=WznV1yfrTOE&pp=ygUJbXljb3RveGlu	Excluded
28	https://www.youtube.com/watch?v=6AcBUu7lGBA&pp=ygUJbXljb3RveGlu	Excluded
29	https://www.youtube.com/watch?v=_LwQhgN6EMA&pp=ygUJbXljb3RveGlu0gcJCccJAYcqIYzv	Excluded
30	https://www.youtube.com/watch?v=mtyjk0MDyAQ&pp=ygUJbXljb3RveGlu	Included in analysis
31	https://www.youtube.com/watch?v=zcogFjmAZjY&pp=ygUJbXljb3RveGlu	Included in analysis
32	https://www.youtube.com/watch?v=Z33ts-Hx3QE&pp=ygUJbXljb3RveGlu	Included in analysis
33	https://www.youtube.com/watch?v=ipg21vcS_iU&pp=ygUJbXljb3RveGlu	Included in analysis
34	https://www.youtube.com/watch?v=LV5pQ6dBvDg&pp=ygUJbXljb3RveGlu	Excluded
35	https://www.youtube.com/watch?v=Z2ajMuwq89M&pp=ygUJbXljb3RveGlu	Excluded
36	https://www.youtube.com/watch?v=t-7YMrPnDuA&pp=ygUJbXljb3RveGlu	Included in analysis
37	https://www.youtube.com/watch?v=VFjWTn-UkdA&pp=ygUJbXljb3RveGlu	Excluded
38	https://www.youtube.com/watch?v=0NSkgKWQcRQ&pp=ygUJbXljb3RveGlu	Included in analysis
39	https://www.youtube.com/watch?v=DUKZ4fgQMds&pp=ygUJbXljb3RveGlu	Included in analysis
40	https://www.youtube.com/watch?v=Jgu0jlET2hg&pp=ygUJbXljb3RveGlu	Included in analysis
41	https://www.youtube.com/watch?v=2iX8CJUp_G8&pp=ygUJbXljb3RveGlu	Included in analysis
42	https://www.youtube.com/watch?v=uo6yeOFdAcQ&pp=ygUJbXljb3RveGlu	Included in analysis

43	https://www.youtube.com/watch?v=Y25UmSZVzys&pp=ygUJbXljb3RveGlu	Excluded
44	https://www.youtube.com/watch?v=JTDodhDYuqI&pp=ygUJbXljb3RveGlu	Excluded
45	https://www.youtube.com/watch?v=sT47MHnPc40&pp=ygUJbXljb3RveGlu	Included in analysis
46	https://www.youtube.com/watch?v=vwXsJaFjbXg&pp=ygUJbXljb3RveGlu	Included in analysis
47	https://www.youtube.com/watch?v=mJzqGk8qFJs&pp=ygUJbXljb3RveGlu	Included in analysis
48	https://www.youtube.com/watch?v=maU7jXf6Rd0&pp=ygUJbXljb3RveGlu	Excluded
49	https://www.youtube.com/watch?v=_7Nj1JOp2ik&pp=ygUJbXljb3RveGlu	Included in analysis
50		No stable standalone watch URL
51	https://www.youtube.com/watch?v=dob8ejbaWHA&pp=ygUJbXljb3RveGlu	Excluded
52	https://www.youtube.com/watch?v=SXZDNAfCYb0&pp=ygUJbXljb3RveGlu	Included in analysis
53	https://www.youtube.com/watch?v=BVisg-URJik&pp=ygUJbXljb3RveGlu	Included in analysis
54	https://www.youtube.com/watch?v=Ja7efoNnUVI&pp=ygUJbXljb3RveGlu	Excluded
55	https://www.youtube.com/watch?v=pX_0xkAdVr0&pp=ygUJbXljb3RveGlu	Included in analysis
56	https://www.youtube.com/watch?v=rrhsl-sNrus&pp=ygUJbXljb3RveGlu	Included in analysis
57	https://www.youtube.com/watch?v=toqR3WScGEA&pp=ygUJbXljb3RveGlu	Excluded
58	https://www.youtube.com/watch?v=ayY9cwsT0uo&pp=ygUJbXljb3RveGlu	Included in analysis
59	https://www.youtube.com/watch?v=X-0pndb9SxQ&list=RDX-0pndb9SxQ&start_radio=1&pp=ygUJbXljb3RveGluoAcB	Excluded
60	https://www.youtube.com/watch?v=oVZt5fm8u8Y&pp=ygUJbXljb3RveGlu	Included in analysis
61	https://www.youtube.com/watch?v=RarWTvDQRT8&pp=ygUJbXljb3RveGlu	Excluded
62	https://www.youtube.com/watch?v=hJ4Gmv6G9LU&pp=ygUJbXljb3RveGlu	Excluded
63	https://www.youtube.com/watch?v=y7Xg-QMSn8E&pp=ygUJbXljb3RveGlu	Included in analysis
64	https://www.youtube.com/watch?v=syRJvupPMW8&pp=ygUJbXljb3RveGlu	Included in analysis
65	https://www.youtube.com/watch?v=Vrd7YUjR81M&pp=ygUJbXljb3RveGlu	Included in analysis
66	https://www.youtube.com/watch?v=06yJ9xDabFM&pp=ygUJbXljb3RveGlu	Included in analysis
67	https://www.youtube.com/watch?v=LjsrD3eO55c&pp=ygUJbXljb3RveGlu	Included in analysis
68	https://www.youtube.com/watch?v=qxCbfz6n_bM&pp=ygUJbXljb3RveGlu	Excluded
69	https://www.youtube.com/watch?v=65SpIu1SRXs&pp=ygUJbXljb3RveGlu	Excluded
70	https://www.youtube.com/watch?v=CdnH11eqsB4&pp=ygUJbXljb3RveGlu	Included in analysis
71	https://www.youtube.com/watch?v=dOhUtKnN2l0&pp=ygUJbXljb3RveGlu	Excluded
72	https://www.youtube.com/watch?v=NADUmlpbI9w&pp=ygUJbXljb3RveGlu	Included in analysis
73	https://www.youtube.com/watch?v=qcRBK0KO1XE&pp=ygUJbXljb3RveGlu	Included in analysis
74	https://www.youtube.com/watch?v=rRlS2ZzbRZE&pp=ygUJbXljb3RveGlu	Excluded
75	https://www.youtube.com/watch?v=YDDr_Z0j0Y0&pp=ygUJbXljb3RveGlu0gcJCccJAYcqIYzv	Included in analysis
76	https://www.youtube.com/watch?v=lcsas5zCma4&pp=ygUJbXljb3RveGlu	Included in analysis
77	https://www.youtube.com/watch?v=NE7EyRI1aAg&pp=ygUJbXljb3RveGlu	Excluded
78	https://www.youtube.com/watch?v=L1319tg5ct4&pp=ygUJbXljb3RveGlu	Included in analysis
79	https://www.youtube.com/watch?v=pk_HiEQnsoY&pp=ygUJbXljb3RveGlu	Included in analysis
80	https://www.youtube.com/watch?v=yX5KCHlEymU&pp=ygUJbXljb3RveGlu	Included in analysis
81	https://www.youtube.com/watch?v=uRUtJ4S7MMQ&pp=ygUJbXljb3RveGlu	Excluded
82	https://www.youtube.com/watch?v=nMrvA39Xcck&pp=ygUJbXljb3RveGlu	Included in analysis
83	https://www.youtube.com/watch?v=X6t1yEwYHmY&pp=ygUJbXljb3RveGlu	Excluded
84	https://www.youtube.com/watch?v=i4rOAx5THlA&pp=ygUJbXljb3RveGlu0gcJCccJAYcqIYzv	Excluded
85	https://www.youtube.com/watch?v=xiFPROMizBM&pp=ygUJbXljb3RveGlu	Excluded
86	https://www.youtube.com/watch?v=C67570-_K3o&pp=ygUJbXljb3RveGlu	Included in analysis

87	https://www.youtube.com/watch?v=C67570-_K3o&pp=ygUJbXljb3RveGlu	Excluded
88	https://www.youtube.com/watch?v=BEZikqngCxc&pp=ygUJbXljb3RveGlu	Included in analysis
89	https://www.youtube.com/watch?v=ZCauPu_kWKo&pp=ygUJbXljb3RveGlu	Included in analysis
90	https://www.youtube.com/watch?v=-UVeD7_k6EU&pp=ygUJbXljb3RveGlu	Included in analysis
91	https://www.youtube.com/watch?v=HWyUy86D2L8&pp=ygUJbXljb3RveGlu	Included in analysis
92	https://www.youtube.com/watch?v=eLPPc3wF1rY&pp=ygUJbXljb3RveGlu	Included in analysis
93	https://www.youtube.com/watch?v=fANOxBu-lFI&pp=ygUJbXljb3RveGlu	Included in analysis
94	https://www.youtube.com/watch?v=wOHjRoxgg_g&pp=ygUJbXljb3RveGlu	Included in analysis
95	https://www.youtube.com/watch?v=FXej_lmBS54&pp=ygUJbXljb3RveGlu0gcJCccJAYcqIYzv	Included in analysis
96	https://www.youtube.com/watch?v=I35UWdz65_Y&t=482s&pp=ygUJbXljb3RveGlu	Included in analysis
97	https://www.youtube.com/watch?v=kP1Zy5tVJIY&pp=ygUJbXljb3RveGlu	Included in analysis
98	https://www.youtube.com/watch?v=AXESIFTjIJA&pp=ygUJbXljb3RveGlu	Included in analysis
99	https://www.youtube.com/watch?v=G8_pM_yqaSE&pp=ygUJbXljb3RveGlu	Included in analysis
100	https://www.youtube.com/watch?v=EuqIsw11S4Y&pp=ygUJbXljb3RveGlu	Excluded
101	https://www.youtube.com/watch?v=aXjMVrYp1fg&pp=ygUJbXljb3RveGlu	Included in analysis
102	https://www.youtube.com/watch?v=g05gZga6bbs&pp=ygUJbXljb3RveGlu	Included in analysis
103	https://www.youtube.com/watch?v=UiF2RrD0p_E&pp=ygUJbXljb3RveGlu	Included in analysis
104	https://www.youtube.com/watch?v=Wru21Twj6jM&pp=ygUJbXljb3RveGlu	Included in analysis
105	https://www.youtube.com/watch?v=LWV03_a_fA&pp=ygUJbXljb3RveGlu	Included in analysis
106	https://www.youtube.com/watch?v=jLagXsvp1fo&pp=ygUJbXljb3RveGlu	Included in analysis
107	https://www.youtube.com/watch?v=SX_zmbk_a3E&pp=ygUJbXljb3RveGlu	Excluded
108	https://www.youtube.com/watch?v=1WDR3fx5CBc&pp=ygUJbXljb3RveGlu	Included in analysis
109	https://www.youtube.com/watch?v=34xpPos07Eo&pp=ygUJbXljb3RveGlu	Included in analysis
110	https://www.youtube.com/watch?v=kuxSTQrilxg&pp=ygUJbXljb3RveGlu	Included in analysis
111	https://www.youtube.com/watch?v=E0z3lSpSdvM&pp=ygUJbXljb3RveGlu	Excluded
112	https://www.youtube.com/watch?v=m3CH3I26w5g&pp=ygUJbXljb3RveGlu	Included in analysis
113	https://www.youtube.com/watch?v=z1uRr_EDhZE&pp=ygUJbXljb3RveGlu0gcJCccJAYcqIYzv	Excluded
114	https://www.youtube.com/watch?v=QHOY9SbQsj0&pp=ygUJbXljb3RveGlu	Included in analysis
115	https://www.youtube.com/watch?v=HUI8nBjkSCA&pp=ygUJbXljb3RveGlu	Included in analysis
116	https://www.youtube.com/watch?v=jhKF_aNUsyY&pp=ygUJbXljb3RveGlu	Excluded
117	https://www.youtube.com/watch?v=k-oW5lEGqdg&pp=ygUJbXljb3RveGlu	Included in analysis
118	https://www.youtube.com/watch?v=rLXzftZ1oyU&pp=ygUJbXljb3RveGlu	Included in analysis
119	https://www.youtube.com/watch?v=kXjHECzjfHA&pp=ygUJbXljb3RveGlu	Excluded
120	https://www.youtube.com/watch?v=0ysl-uGEMkM&pp=ygUJbXljb3RveGlu	Excluded
121	https://www.youtube.com/watch?v=yfZOtrzCbs8&pp=ygUJbXljb3RveGlu	Included in analysis
122	https://www.youtube.com/watch?v=mQ77aSf7QMQ&pp=ygUJbXljb3RveGlu	Included in analysis
123	https://www.youtube.com/watch?v=GXweTx4PFoc&pp=ygUJbXljb3RveGlu	Excluded
124	https://www.youtube.com/watch?v=ypV4fV3nldw&pp=ygUJbXljb3RveGlu	Included in analysis
125	https://www.youtube.com/watch?v=sc-2Zu4kM7w&pp=ygUJbXljb3RveGlu	Included in analysis
126	https://www.youtube.com/watch?v=dz89X9wcZ3Y&pp=ygUJbXljb3RveGlu	Excluded
127	https://www.youtube.com/watch?v=izmQt5GDGOc&pp=ygUJbXljb3RveGlu	Included in analysis
128	https://www.youtube.com/watch?v=dTMjPZkgnm4&pp=ygUJbXljb3RveGlu	Included in analysis
129	https://www.youtube.com/watch?v=FpheRy9f9zE&pp=ygUJbXljb3RveGlu	Included in analysis

130	https://www.youtube.com/watch?v=e6PiIBXNVEI&pp=ygUJbXljb3RveGlu0gcJCccJAYcqIYzv	Included in analysis
131	https://www.youtube.com/watch?v=m-NUQbA7DDA&pp=ygUJbXljb3RveGlu	Excluded
132	https://www.youtube.com/watch?v=SX_zmbk_a3E&pp=ygUJbXljb3RveGlu	Excluded
133	https://www.youtube.com/watch?v=6IKc-aJwyzQ&pp=ygUJbXljb3RveGlu	Included in analysis
134	https://www.youtube.com/watch?v=JqLEPXYO6zA&pp=ygUJbXljb3RveGlu	Included in analysis
135		No stable standalone watch URL
136	https://www.youtube.com/watch?v=b1S5ZPtj6PI&pp=ygUJbXljb3RveGlu	Excluded
137	https://www.youtube.com/watch?v=M4d5iuDfMFI&pp=ygUJbXljb3RveGlu	Included in analysis
138	https://www.youtube.com/watch?v=KjMzvgDR3CI&list=RDKjMzvgDR3CI&start_radio=1&pp=ygUJbXljb3RveGluoAcB	Excluded
139	https://www.youtube.com/watch?v=OZbzQN4DnfI&pp=ygUJbXljb3RveGlu	Included in analysis
140	https://www.youtube.com/watch?v=nD_lxUjamXg&pp=ygUJbXljb3RveGlu	Included in analysis
141	https://www.youtube.com/watch?v=y3U4iqUBhPY&pp=ygUJbXljb3RveGlu	Included in analysis
142	https://www.youtube.com/watch?v=tkbOwH7fss8&pp=ygUJbXljb3RveGlu	Included in analysis
143	https://www.youtube.com/watch?v=IfVNO4Hw2Lw&pp=ygUJbXljb3RveGlu	Included in analysis
144		No stable standalone watch URL
145	https://www.youtube.com/watch?v=YbT7RseXPd0&pp=ygUJbXljb3RveGlu	Included in analysis
146	https://www.youtube.com/watch?v=lD8SdNCMMfE&pp=ygUJbXljb3RveGlu0gcJCccJAYcqIYzv	Included in analysis
147	https://www.youtube.com/watch?v=eRqlzPGpPGA&pp=ygUJbXljb3RveGlu	Included in analysis
148	https://www.youtube.com/watch?v=CXqAiWfNvL0&pp=ygUJbXljb3RveGlu	Included in analysis
149	https://www.youtube.com/watch?v=Gdelges1i-8&pp=ygUJbXljb3RveGlu	Included in analysis
150	https://www.youtube.com/watch?v=XdiF9JbbYaY&pp=ygUJbXljb3RveGlu	Included in analysis
151	https://www.youtube.com/watch?v=CuBMBwb0uMk&pp=ygUJbXljb3RveGlu	Included in analysis
152	https://www.youtube.com/watch?v=Mn0r20ui9I0&pp=ygUJbXljb3RveGlu0gcJCccJAYcqIYzv	Included in analysis
153	https://www.youtube.com/watch?v=moClzxNQNVg&pp=ygUJbXljb3RveGlu0gcJCccJAYcqIYzv	Excluded
154	https://www.youtube.com/watch?v=-68pKozZPmU&pp=ygUJbXljb3RveGlu	Included in analysis
155	https://www.youtube.com/watch?v=5ZYY_X3swYc&pp=ygUJbXljb3RveGlu	Included in analysis
156	https://www.youtube.com/watch?v=TTu58R2J5I0&pp=ygUJbXljb3RveGlu	Included in analysis
157	https://www.youtube.com/watch?v=-Y-dIZwgCtg&pp=ygUJbXljb3RveGlu	Included in analysis
158	https://www.youtube.com/watch?v=8wqXRa91oHs&pp=ygUJbXljb3RveGlu	Included in analysis
159	https://www.youtube.com/watch?v=j_4qQfnD9II&pp=ygUJbXljb3RveGlu	Included in analysis
160	https://www.youtube.com/watch?v=UPxwZCo7ZoI&pp=ygUJbXljb3RveGlu	Included in analysis
161	https://www.youtube.com/watch?v=RxNAuk5k_Jg&pp=ygUJbXljb3RveGlu	Included in analysis
162		No stable standalone watch URL
163	https://www.youtube.com/watch?v=EnOLzvjofFM&pp=ygUJbXljb3RveGlu	Included in analysis
164	https://www.youtube.com/watch?v=vXuIrRlRIWg&pp=ygUJbXljb3RveGlu	Included in analysis
165	https://www.youtube.com/watch?v=5IjFP23BRlM&pp=ygUJbXljb3RveGlu0gcJCccJAYcqIYzv	Included in analysis
166	https://www.youtube.com/watch?v=8UXslPDk5X8&pp=ygUJbXljb3RveGlu	Excluded
167	https://www.youtube.com/watch?v=W1MF4LBe_PY&pp=ygUJbXljb3RveGlu	Included in analysis
168	https://www.youtube.com/watch?v=O7QvVTAAXNA&pp=ygUJbXljb3RveGlu	Included in analysis
169	https://www.youtube.com/watch?v=iyrRxQjiiZM&pp=ygUJbXljb3RveGlu	Included in analysis
170	https://www.youtube.com/watch?v=W3hHfup5Cuo&pp=ygUJbXljb3RveGlu	Included in analysis
